# p-Aminobenzene-Sulfonamide Derivatives of Substituted Pyrimidines as Human Carbonic Anhydrase Inhibitors

**DOI:** 10.3390/ijms27062725

**Published:** 2026-03-17

**Authors:** Andrea Angeli, Anthi Petrou, Victor Kartcev, Mikhail Prezent, Samvel Sirakanyan, Athina Geronikaki, Claudiu T. Supuran

**Affiliations:** 1 Neuro Farba Department, Sezione di Scienze Farmaceutiche, Università degli Studi di Firenze, Via Ugo Schiff 6, 50019 Sesto Fiorentino, Italyclaudiu.supuran@unifi.it (C.T.S.); 2 School of Health, Department of Pharmacy, Aristotle University of Thessaloniki, 54124 Thessaloniki, Greece; 3 InterBioscreen, 11991 Moscow, Russia; 4 Zelinsky Institute of Organic Chemistry, Leninsky Prospect, 11991 Moscow, Russia; 5 Scientific Technological Center of Organic and Pharmaceutical Chemistry, National Academy of Science of Republic of Armenia, Institute of Fine Organic Chemistry of A. L. Mnjoyan, 26, Azatutian Ave., Yerevan 0014, Armenia

**Keywords:** benzenesulfonamides, carbonic anhydrase, heterocyclic, inhibition, docking

## Abstract

The essential reaction of CO_2_ hydration, fundamental to all living organisms, is facilitated by the enzyme carbonic anhydrase (CA, EC 4.2.1.1). This enzyme plays a crucial role in regulating various physiological and pathological processes. A series of heterocyclic benzenesulfonamide derivatives (19 compounds) were evaluated as possible inhibitors of human CAs. Their inhibitory properties were tested against several isoforms such as the cytosolic hCA I and hCA II, as well as the transmembrane isoforms hCA IV, hCA IX and hCA XII. The tested molecules demonstrated notable inhibitory potential, particularly toward hCA II and hCA IV, where five and four compounds, respectively, exhibited greater potency than the reference inhibitor, acetazolamide. Molecular docking simulations were further performed to elucidate the binding interactions of the most active compounds with the human CA II, IV IX and XII isoforms

## 1. Introduction

Carbonic anhydrase (CA) refers to a family of zinc metalloenzymes that catalyze the reversible hydration of carbon dioxide to bicarbonate and protons. Multiple classes of CAs exist, distinguished by their structural and genetic characteristics and classified as α-, β-, γ-, η-, δ-, ζ-, θ-, and ι-Cas [1]. Among them, α-CAs are found in plants, algae, and vertebrates, and the crystallographic data for all isoforms have been determined and deposited in the Protein Data Bank (PDB) [2].

In humans, 16 CA isoforms have been identified and categorized according to their subcellular localization and catalytic activity: CA I, CA II, CA III, CA VII, and CA XIII are cytosolic [3,4]; CA IV, CA IX, CA XII, CA XIV, and CA XV are membrane-associated [5,6]; CA VA and CA VB are mitochondrial [5,6]; and CA VI is secreted in saliva and colostrum [2]. Three isoforms, CA VIII, CA X, and CA XI, are catalytically inactive and are collectively known as carbonic anhydrase-related proteins (CARPs). Among the catalytically active isoforms, hCA II is the most extensively studied due to its high catalytic efficiency, abundance, and ease of crystallization.

CAs play vital roles in various physiological processes, including acid–base balance regulation, CO_2_ transport, respiration, bone resorption, and tumorigenesis. Dysregulation or overexpression of specific CA isoforms is associated with several diseases. Pharmacologically, both inhibition and activation of CAs have therapeutic implications. For instance, inhibitors targeting hCA XIV, hCA XII, hCA IV, and hCA II are clinically employed as diuretics [7]. For example, elevated levels of hCA I have been associated with cerebral and retinal edema, while hCA II overexpression has been linked to conditions such as altitude sickness, edema, epilepsy, and glaucoma. Similarly, increased expression of hCA IX is frequently observed in cancer, whereas hCA IV has been implicated in glaucoma, retinitis pigmentosa, retinal disorders, and stroke [8]. The involvement of these enzymes in diverse disease states highlights their significance as promising therapeutic targets. Moreover, given the high degree of similarity in the active-site structures among the twelve catalytically active hCA isoforms [9,10], the development of isoform-selective inhibitors is crucial to minimize off-target effects and to enable the design of safer and more effective therapeutic agents.

Among the various hCA isoforms, hCA IX and hCA XII are primarily associated with malignant tissues and exhibit minimal expression in healthy cells. Both enzymes are multidomain transmembrane proteins characterized by a catalytically active carbonic anhydrase domain located on the extracellular side of the membrane. The hCA IX isoform has become a major focus of research interest, prompting extensive efforts toward the design and development of highly effective hCA IX inhibitors. A wide range of inhibitory molecules have been described in the literature, with most compounds falling into chemical classes such as sulfonamides, dithiocarbamates, coumarins, sulfocoumarins, sulfamates, and carboxylates.

Currently, several compounds have been identified as effective hCA IX inhibitors, with activity in the low nanomolar or subnanomolar range; however, in addition to inhibitory potency, a critical parameter for the therapeutic development of these compounds is their selectivity toward individual hCA isoforms. Particular attention must be given to minimizing activity against hCA I and hCA II, which are widely expressed and play essential physiological roles [11]. This consideration is especially relevant for hCA II, as it exhibits the broadest tissue distribution and is abundantly present in erythrocytes. Because most carbonic anhydrase inhibitors are administered systemically and readily cross cell membranes, non-selective compounds are prone to binding hCA II, leading to drug sequestration in red blood cells [12]. This phenomenon can lower systemic drug levels, reduce bioavailability for hCA IX, and ultimately limit drug accumulation within tumor tissues [13]. Consequently, achieving isoform selectivity remains the primary challenge in the development of clinically effective hCA inhibitors. The difficulty in identifying isoform-specific agents stems from the extensive sequence similarity and strong structural conservation shared across the hCA family.

Pyrimidine derivatives have attracted considerable attention due to their broad spectrum of biological and pharmacological activities, including anticancer [11,12,13], anti-inflammatory [14,15], antioxidant [14,16], COX-2 inhibitory [17], antiulcer [17], antimicrobial [18,19], antidiabetic [20], antiviral [21,22], anti-HIV [23], and carbonic anhydrase inhibitory properties [24,25,26,27,28,29,30]. Korkusuz et al. [25] synthesized novel pyrimidine derivatives that exhibited Ki values ranging from 39.16 ± 7.70 to 144.62 ± 26.98 nM against hCA I and 18.21 ± 3.66 to 136.35 ± 21.48 nM against hCA II, both of which are implicated in diverse physiological and pathological processes. These compounds also showed inhibitory activity against AChE (33.15 ± 4.85–52.98 ± 19.86 nM) and BChE (31.96 ± 8.24–69.57 ± 21.27 nM).

Similarly, Manzoor et al. [24] designed and synthesized a series of triazole–sulfonamide-bearing pyrimidine derivatives via click chemistry and evaluated them against hCA I, II, IX, and XII isoforms. Most of the compounds demonstrated potent inhibitory activity toward hCA II and moderate inhibition of hCA I, with several exhibiting remarkable potency (Ki = 0.82–9.4 nM) and selectivity toward tumor-associated hCA XII compared to hCA IX.

Considering these findings, the present study focuses on the evaluation of CA inhibitory activity against hCA I, hCA II, hCA IV, hCA IX, and hCA XII isoforms.

## 2. Results and Discussion

**Scheme 1:**
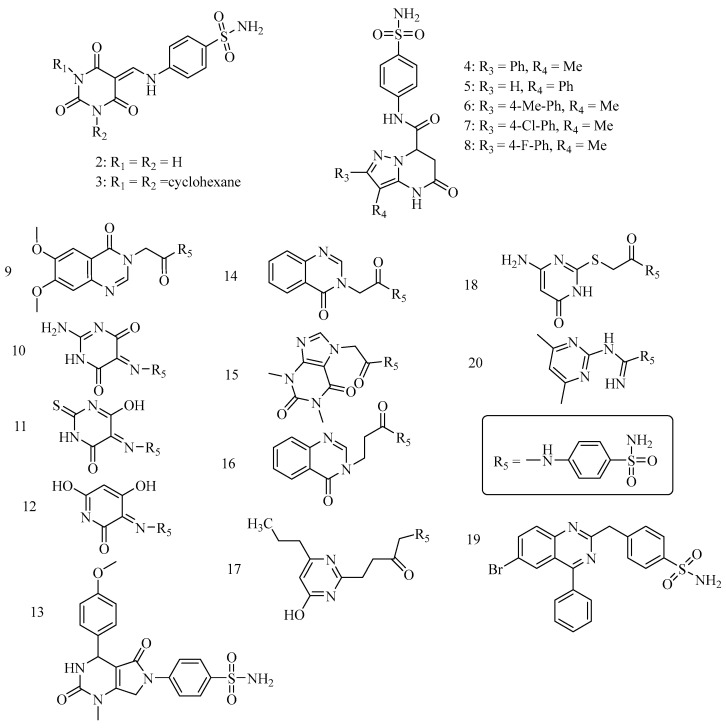
Structures of compounds tested.

**Scheme 2:**
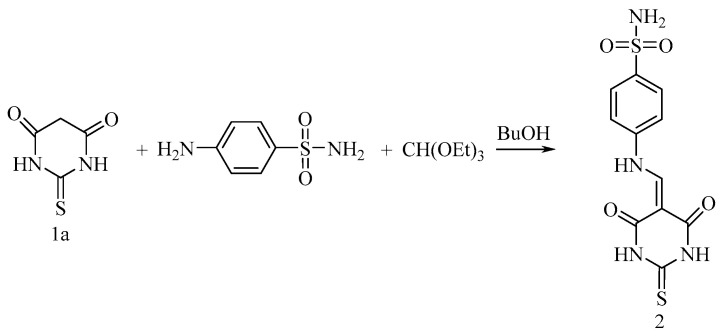
Synthesis of 4-[[(tetrahydro-4,6-dioxo-2-thioxo-5(2H)-pyrimidinylidene)methyl]amino]benzenesulfonamide **2**.

In this paper we report the synthesis of two compounds, and their structures are presented in Scheme 1. The structural assignments were confirmed by 1H NMR spectroscopy. The proposed synthetic schemes (and references for the preparation of homologues) for the remaining 18 compounds are presented in the Supplementary Material. 4-[[(Tetrahydro-4,6-dioxo-2-thioxo-5(2H)-pyrimidinylidene)methyl] amino] benzen esulfonamide **2** was obtained according to the procedure described in the article (Scheme 2). The structure was confirmed by ^1^H;^13^C NMR spectra as well as mass spectra.

**Scheme 3:**
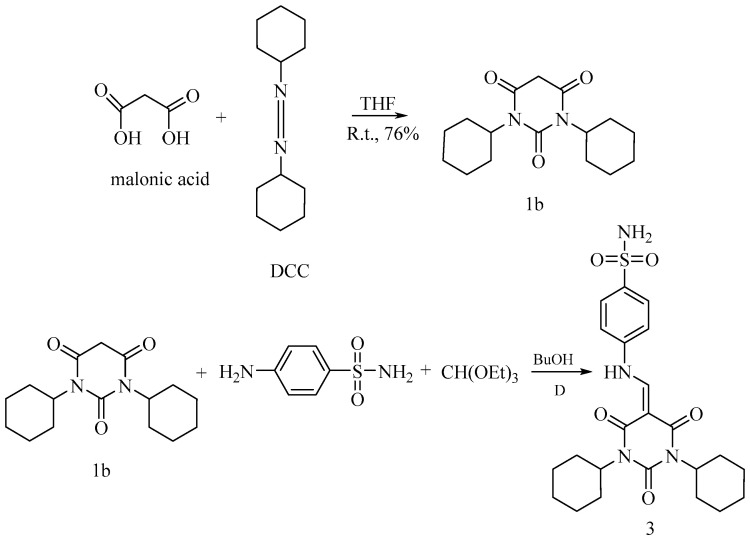
Synthesis of 4-{[(1,3-dicyclohexyl-2,4,6-trioxotetrahydro-5(2H)-pyrimidinylidene)methyl]amino}benzenesulfonamide **3**.

Dicyclohexyl-2,4,6-trioxotetrahydro-5(2H)-pyrimidinylidene)methyl]amino}benzene-sulfonamide **3** was prepared following the reaction in Scheme 3.

The rest of the compounds were obtained from InterBioScreen for the evaluation of CA inhibitory activity based on PASS prediction.

### 2.1. In Vitro Inhibition of Carbonic Anhydrase Isoforms

**Figure 1:**
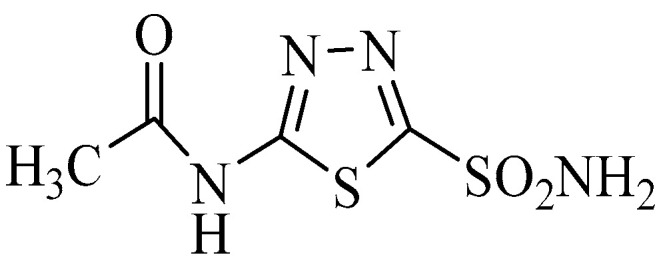
Structure of acetazolamide (AAZ).

The inhibitory potential of all compounds (**19**) was evaluated against five human CA isoforms, namely hCA I, hCA II, hCA IV, hCA IX, and hCA XII, using acetazolamide (AAZ) as a reference drug (Figure 1), and the results are shown in Scheme 1.

According to the data presented in Table 1, all tested compounds demonstrated inhibitory activity against the evaluated human carbonic anhydrase (hCA) isoforms. However, their potency and selectivity varied significantly across the five isoforms studied. Notably, several compounds exhibited stronger inhibition than the reference drug acetazolamide (AAZ) against hCA I, hCA II, and hCA IV.

The Ki values of compounds toward cytosolic enzyme hCA I ranged from 65.7 to 8604 nM. Among them, compound **4**, featuring a 2-(phenyl)-3-methyl-6,7-dihydropyrazolo [1,5-a]pyrimidin-5(4*H*)-one scaffold, showed the strongest inhibition (Ki = 65.7 nM), outperforming AAZ (Ki = 250 nM). Compound **8**, which differs from compound **4** by the presence of an electron-withdrawing fluorine atom at the para position of the benzene ring, exhibited slightly reduced activity (Ki = 78.7 nM), likely due to this substitution.

**Figure 2:**
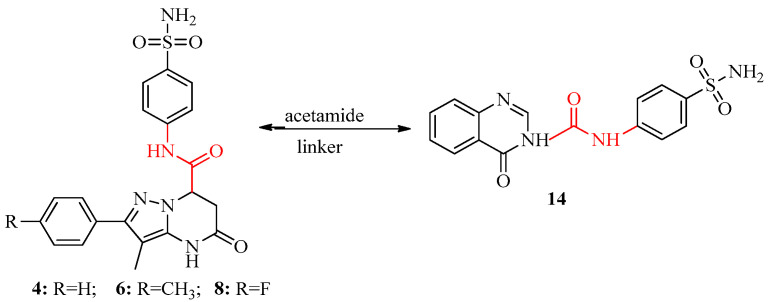
Structures of the most active compounds towards the hCA I isoform.

Compounds **6** and **14** demonstrated comparable potency to that of AAZ, with Ki values of 268 nM and 266.5 nM, respectively (Figure 2). In contrast, compound **3**, which contains bulky dicyclohexyl substituents, showed the weakest inhibition (Ki = 8604 nM), likely due to steric hindrance.

Against hCA II, the tested compounds displayed markedly improved activity, with Ki values ranging from 3.6 to 926.9 nM. Several compounds, including **7**, **8**, **10**, **13**, and **14**, exhibited stronger inhibition than AAZ (Ki = 12 nM), with Ki values of 7.0, 6.1, 6.9, 8.3, and 3.6 nM, respectively. All of these compounds have common structural elements, namely pyrimidin-dion or tetrahydropyrimidin-dion moieties linked through various linkers to a benzenesulfonamide ring.

Structure–activity relationship (SAR) analysis revealed that compound **14**, the most potent (Ki = 3.6 nM), likely owes its high activity to its smaller molecular size, which facilitates better access to the active site. Compound **8** (Ki = 6.1 nM), containing a 4-fluorophenyl group, was more active than compound **7** (Ki = 7.0 nM), which bears a 4-chlorophenyl substituent, indicating that fluorine slightly enhances activity compared to chlorine. Both compounds contain a pyrimidinedione ring fused to a 3-(4-substituted phenyl)-4-methylpyrazole scaffold. Compound **10**, which incorporates a 2-aminopyrimidine-dione moiety connected to the benzenesulfonamide core via a hydrazine linker, showed similar activity to compound **7**.

**Figure 3:**
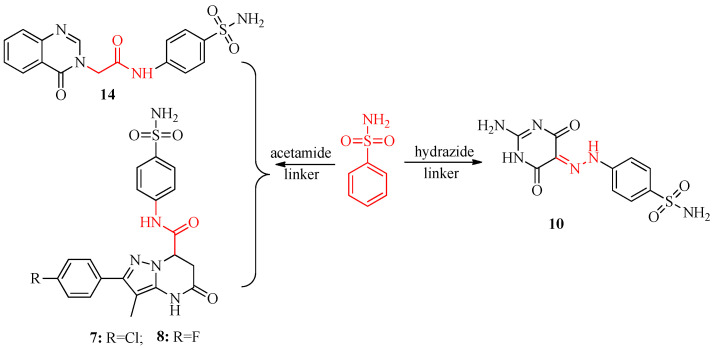
Structures and linkers of the most active compounds against hCA II and hCA IV isoforms.

**Figure 4:**
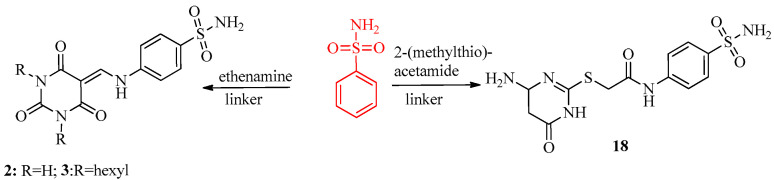
Structures with linkers of less active compounds against the hCA II isoform.

In contrast, compounds **2**, **3**, **18**, and **19** showed the lowest activity against hCA II (Figure 3). Comparison of these weakly active compounds with the highly active analogues revealed that compounds **2**, **3**, and **18** share a structural element with the active compounds **7** and **8**. However, in the active series this feature is incorporated into a fused tetrahydropyrimidine-dione/3-(4-substituted phenyl)-4-methyl-pyrazole scaffold. Another key structural difference is the nature of the linker: the active compounds contain an acetamide linker, whereas the inactive analogues contain an ethanimine linker. The increased flexibility of the acetamide moiety likely facilitates better accommodation within the enzyme’s active site compared to the more rigid ethanimine linker (Figure 4).

Compounds such as **19** (6-bromo-4-phenylquinazoline) and **2** (a 2-thion-substituted dihydropyrimidine-dione), both connected to the benzenesulfonamide core via a hydrazine linker, also exhibited weak inhibition, further indicating that these structural features are unfavorable for hCA II activity.

For the membrane-bound isoform hCA IV, Ki values ranged from 5.4 to 2258 nM. Compounds **7**, **8**, and **14** exhibited considerably stronger inhibition than AAZ (Ki = 74 nM), with Ki values of 28.5, 5.4, and 29.5 nM, respectively. As observed for hCA I, compound **8** was the most potent, with a selectivity index (SI) of 14.5 (Figure 4).

Replacing the fluorine atom in compound **8** with chlorine (compound **7**) led to a notable decrease in activity (Ki = 28.5 nM). Substituting the dihydropyrazolo [1,5-a]pyrimidinone core with a quinazolinone ring (compound **14**) resulted in a slight reduction in activity. Furthermore, removal of the chlorine substituent (compound **7**) or repositioning the benzene ring (compounds **4** and **5**) also decreased activity, highlighting the importance of both the nature and position of the substituent on the aromatic ring.

**Figure 5:**
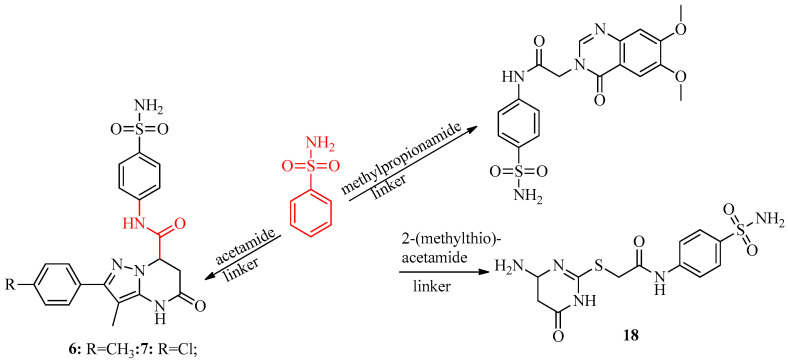
Structure of compounds and their linkers with activity equal to AAZ.

In general, the compounds were less potent against the tumor-associated isoform hCA IX, with Ki values ranging from 30.3 to 162.2 nM. However, compounds **6**, **7**, **9**, and **18** exhibited inhibitions comparable to AAZ (Ki = 25.8 nM), with compound **9** being the most active (Ki = 30.3 nM), followed by compound **18** (Ki = 32.4 nM). Replacing the 6,7-dimethoxyquinazolinone core with the dihydropyrazolopyrimidinone moiety, as observed in compounds **6** and **7**, resulted in slightly reduced activity (Figure 5).

Some compounds also demonstrated isoform selectivity, particularly toward hCA I and IV. For example, compound **2** exhibited selectivity index (SI) values of 73 for hCA I and 18 for hCA IV, whereas compound **3** showed even higher selectivity with SI values of 210 for hCA I and 50 for hCA IV. Compounds **4**, **5**, and **6** also demonstrated notable selectivity toward hCA IV, with SI = 47, 48, and 37, respectively.

Against hCA XII, Ki values ranged from 7.5 to 89 nM, with no compound surpassing AAZ. However, compounds **2**, **8**, and **11** showed high activity with Ki values of 8.5, 8.7, and 8.6 nM, respectively. Substitution of one oxygen atom in the pyrimidine-trione core with sulfur did not significantly affect activity. Similarly, replacing the trione core with a dihydropyrazolopyrimidinone moiety also had minimal impact on activity.

Overall, the tested compounds exhibited the strongest inhibition against hCA II, followed by hCA IV. The rank order of isoform inhibition potency was: hCA II > hCA IV > hCA I > hCA XII > hCA IX. While inhibition of hCA XII was generally lower than of hCA I and IV, several compounds showed notable selectivity toward hCA I and IV, highlighting their potential for isoform-specific therapeutic targeting.

### 2.2. Selectivity Parameters of Synthesized Compounds

The human carbonic anhydrase (hCA) isoforms exhibit a high degree of similarity, with their primary amino acid sequences sharing more than 30% identity. Designing inhibitors that selectively target specific hCA isoforms (hCAIs) is challenging because much of this sequence homology involves residues within the active site. However, newly synthesized heterocyclic benzenesulfonamides have demonstrated significant selectivity for the transmembrane isoforms hCA IX and XII, compared to the cytosolic isoforms hCA I and II, as well as the membrane-associated hCA IV. The selectivity of these compounds toward various hCA isoforms was assessed using the enzyme selectivity index (SI), defined as the ratio of the inhibition constant (KI) values for hCA I, II, and IV relative to those for hCA IX and XII. The SI values, summarized in Table 2, were employed to evaluate the comparative enzyme selectivity of the synthesized compounds.

With respect to selectivity for hCA IX compared to the hCA I isoform, the calculated SI (I/IX) values for the synthesized compounds ranged from 0.68 to 83.75. Among them, compound **12**, which contains a 2,6-dihydroxypyrimidin-4(5H)-one fragment, exhibited the highest selectivity toward hCA IX, making it almost 8.6 times more selective than AAZ. In contrast, the presence of 4-(4-methoxyphenyl)-7-methyl-4,5-dihydro-1H-pyrrolo [3,4-c]pyridine-3,6(2*H*,7*H*)-dione and 4,6-dimethylpyrimidine moieties notably decreased the selectivity index.

The selectivity of the tested compounds toward hCA IX compared with the hCA II isoform showed relatively low potency, with calculated SI values ranging from 0.002 to 15.9, the highest value being observed for compound **19**. Almost all compounds except for **16**–**19** were selective towards hCA II. In contrast, when comparing selectivity toward hCA IX versus hCA IV, the SI values varied between 0.14 and 51.44, with compound **19** again exhibiting the greatest selectivity.

In terms of hCA XII selectivity relative to hCA I, the selectivity index (SI) values varied from 1.13 to 944.71, with compound **2** exhibiting the highest selectivity (SI = 944.71). The presence of a 2-thioxodihydropyrimidine-4,6(1*H*,5*H*)-dione group was found to significantly enhance selectivity, whereas the introduction of a 3-methyl-2-phenyl-6,7-dihydropyrazolo [1,5-a]pyrimidin-5(4*H*)-one moiety led to the lowest SI value (1.13). Regarding selectivity for hCA XII relative to hCA II, the SI values ranged from 0.15 to 109.1, with compound **2** once again showing the highest selectivity. But seven compounds (**5**–**8**, **10**, **13**, and **14**) were selective towards hCAII. For selectivity against hCA IV, the SI values ranged from 0.39 to 236, with compound **20** demonstrating the strongest selectivity.

### 2.3. Molecular Docking Studies

To explore the potential inhibition mechanisms of the tested compounds, molecular docking studies were performed on the most active candidates: compounds **2**, **4**, **7**, **8**, **10**, **12** and **14**. The active site of human carbonic anhydrase (hCA) isoforms is structurally conserved, measuring approximately 15 Å in both depth and width. Within this pocket, the key residues His94, His96, and His119 act as zinc ligands, while Thr199 and Glu105 function as critical “gatekeepers” regulating substrate access [31,32,33,34]. Despite these conserved features, variations in the residues surrounding the entrance and exit of the active site contribute to isoform-specific binding interactions.

Table 3 summarizes the molecular docking results for the tested compounds against hCA I, II, IV, and IX isoforms. Docking analysis revealed that all compounds interact with the enzymes by coordinating the Zn(II) ion in their deprotonated anionic state. This coordination primarily occurs through the negatively charged nitrogen of the sulfonamide group, a key feature contributing to their inhibitory activity [35].

Docking studies suggest that the selectivity and inhibitory profiles of the compounds for each isoform are influenced by differences in the enzyme active sites. Specifically, the composition of amino acids in the active site significantly affects the final conformation and interactions of the compounds, ultimately impacting their inhibitory properties. For example, compound **8**, which binds to both hCA IX and hCA II, adopts distinct conformations in each enzyme. This difference may arise from the presence of the hydrophobic residue Phe131 in hCA II, whereas hCA IX contains the smaller residue Val131. The smaller size of Val131 in hCA IX allows ligands such as compound **8** to penetrate deeper into the active site, leading to a different configuration with fewer interactions.

**Figure 6:**
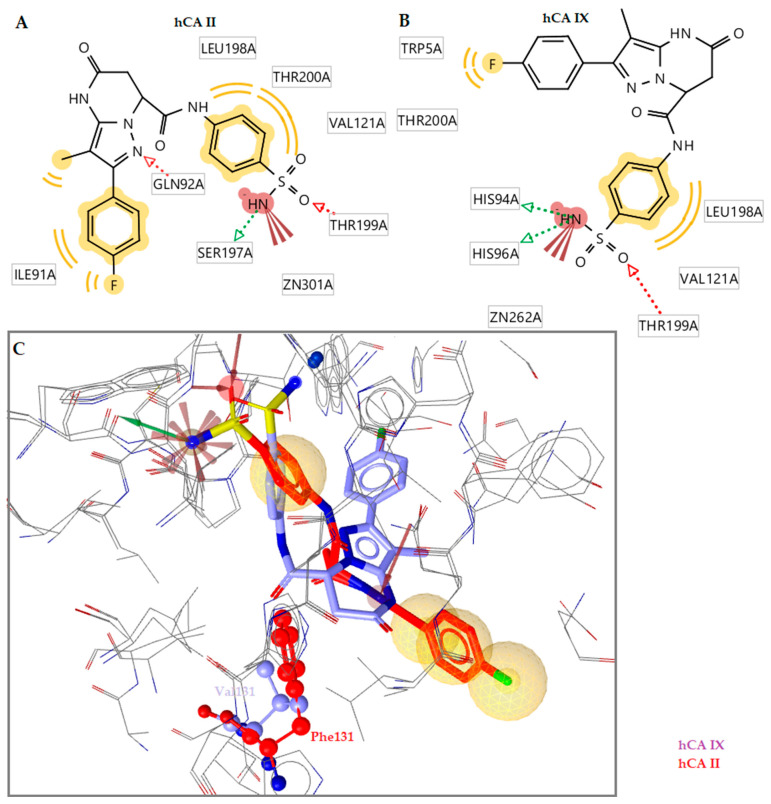
(**A**) 2D interaction diagram of compound **8** docking pose interactions with the key amino acids in hCA II and (**B**) in hCA IX. (**C**) Superposition of compound **8** bound to hCA II (red) in comparison to hCA IX (purple), with specific residues labeled. Active site zinc is shown as blue spheres, red dotted and green arrows indicate H-bonds, and yellow spheres represent hydrophobic interactions.

As illustrated in Figure 1, in both enzyme–ligand complexes, the negatively charged nitrogen of the sulfonamide group coordinates the Zn(II) ion and forms hydrogen bonds. Additionally, hydrophobic interactions are observed in both cases, contributing to the stabilization of the complexes and reducing their binding free energy (Figure 6).

The most active compound, **8**, exhibited strong binding interactions when docked into the hCA I isoform. Specifically, its sulfonamide group formed two stabilizing hydrogen bonds with the backbone of Thr199 and His200, contributing to its high inhibitory potency. Similarly, molecular docking studies of compound **8** with the hCA IV isoform revealed the formation of three hydrogen bonds, in addition to numerous hydrophobic interactions, further reinforcing its strong binding affinity.

**Figure 7:**
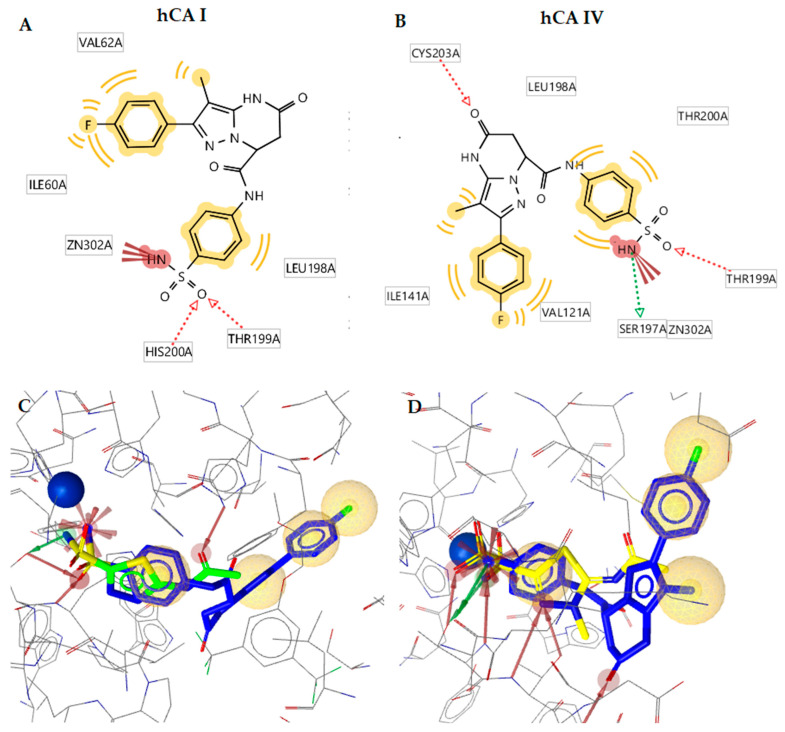
(**A**) 2D interaction diagram of compound **8** docking pose interactions with the key amino acids in hCA I. (**B**) 2D interaction diagram of compound **8** docking pose interactions with the key amino acids in hCA IV. (**C**) Superposition of compound **8** (blue) and AZZ (green) bound to hCA I. (**D**) Superposition of compound **8** (blue) and AZZ (green) bound to hCA IV. Active site zinc is shown as blue spheres, red dotted and green arrows indicate H-bonds, and yellow spheres represent hydrophobic interactions.

A key feature observed in both isoforms was the coordination of the Zn(II) ion by the negatively charged nitrogen of the sulfonamide group. Notably, the structural superposition of compound **8** and the reference inhibitor acetazolamide (AAZ) within the hCA II and IV isoforms demonstrated a critical difference: AAZ was unable to establish a direct interaction with the Zn(II) ion, which likely accounts for its lower inhibitory activity in comparison (Figure 7).

**Figure 8:**
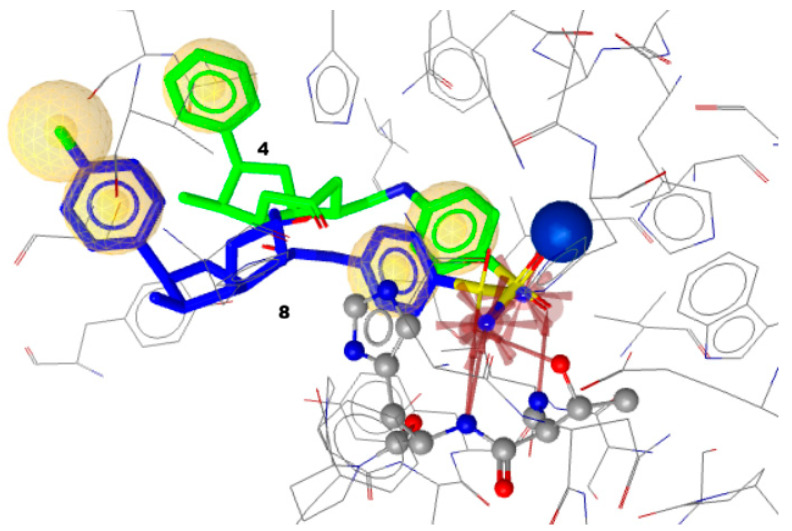
Superposition of compound **8** (blue) and **4** (green) bound to hCA I. Active site zinc is shown as blue spheres, red dotted and green arrows indicate H-bonds, and yellow spheres represent hydrophobic interactions.

In comparison with compound **4**, compound **8** displayed enhanced binding within the hCA I active site (Figure 8). While both compounds exhibited the same key anchoring interactions—namely, the formation of stabilizing hydrogen bonds between the sulfonamide group and the backbone residues Thr199 and His200—compound **8** showed an additional contribution to binding affinity arising from its fluoro substituent on the benzene ring. This fluorine atom enabled an extra interaction with Val62, which was not observed for compound **4**. The presence of this additional contact, together with the conserved hydrogen-bonding network and hydrophobic interactions, rationalizes the higher inhibitory potency of compound **8** relative to compound **4**.

Finally, although compounds **2**, **4** and **12** were able to enter the active site of hCA II, hCA IX, and hCA IV, they did not exhibit significant inhibitory activity against these isoforms because they were unable to effectively reach and coordinate the catalytic Zn^2+^ ion. Molecular docking studies showed that their binding conformations within the active site differed markedly from those of the most active compounds. In particular, the orientation and positioning of the sulfonamide group were unfavorable, preventing proper approach to the zinc center and the formation of the key metal–ligand interactions required for enzyme inhibition. Consequently, despite being accommodated in the catalytic cavity, compounds **2**, **4** and **12** failed to block the active site efficiently, which rationalizes their lack of activity.

### 2.4. Drug-Likeness and Bioavailability Score

The drug-likeness and bioavailability scores of all compounds are summarized in Table 4. One of the most critical challenges in drug development is ensuring efficient oral absorption, as it significantly influences a compound’s therapeutic potential. In this study, the majority of the tested compounds exhibited low gastrointestinal absorption, with the exception of compounds **13**, **14**, and **16**, which demonstrated favorable absorption profiles. Metabolism predictions further revealed that compounds are not inhibitors of the cytochrome P450 enzyme CYP2D6, suggesting a lower likelihood of drug–drug interactions associated with this metabolic pathway. Additionally, toxicity assessments indicated that all compounds, except for compounds **1** and **2**, showed no pain-related indications and could be considered non-toxic.

**Figure 9:**
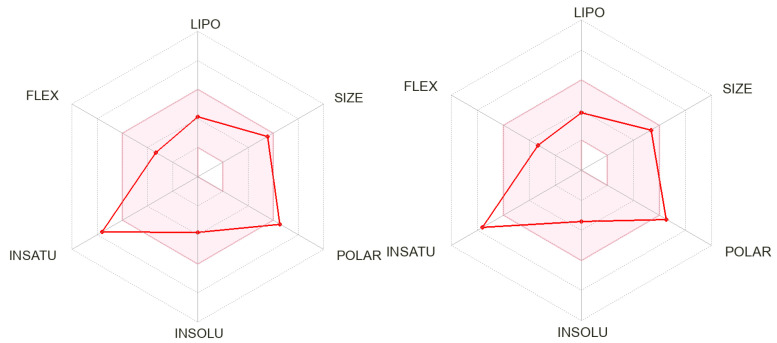
Bioavailability radar plots of compounds **15** and **16**. In the plots, the pink area represents the optimal range for each property for oral bioavailability (lipophilicity (LIPO): XLOGP3 between −0.7 and +5.0; molecular weight (SIZE): MW between 150 and 500 g/mol; polarity (POLAR): TPSA between 20 and 130 Å^2^; solubility (INSOLU): log S not higher than 6; saturation (INSATU): fraction of carbons in the sp^3^ hybridization not less than 0.25; and flexibility (FLEX): no more than nine rotatable bonds).

According to predictive models, all compounds exhibited a bioavailability score of 0.55, with no violations of Lipinski’s Rule of Five, indicating good drug-likeness and oral bioavailability potential. Among the evaluated compounds, those displaying the most promising pharmacokinetic and drug-likeness profiles were compounds **7** and **8**, which achieved drug-likeness scores of 1.47 and 1.33, respectively (Table 4, Figure 9). These findings highlight compounds **15** and **16** as potential lead candidates for further optimization and development.

## 3. Materials and Methods

### 3.1. Chemistry

#### 3.1.1. Synthesis of 4-[[(Tetrahydro-4,6-dioxo-2-thioxo-5(2H)-pyrimidinylidene)methyl]amino]benzenesulfonamide (**2**)

To a hot stirred solution of thiobarbituric acid 1a (0.490 g; 3.4 mmol) in 1-butanol (25 mL) in the presence of slight excess of triethylorthoformate (0.532 g; 3.6 mmol), 4-aminobenzenesulfonamide (0.585 g; 3.4 mmol) was added, and the resulting reaction mixture was heated under reflux for 3 h. The solid formed was collected in hot state by suction filtration to obtain 4-[[(tetrahydro-4,6-dioxo-2-thioxo-5(2H)-pyrimidinylidene)methyl]amino]benzenesulfonamide (**2**) (1.052 g, 95%), m.p. 336–339 °C (decompose). ^1^H (600 MHz, DMSO-d_6_): 7.40 (s, 2H, SO_2_NH_2_), 7.75 (d, *J*= 8.6 Hz, 2H, orto-ArH), 7.83 (d, *J* = 8.6 Hz, 2H, para-ArH), 8.64 (d, *J* = 13.9 Hz, 1H, CH=N), 12.05 (d, *J* = 13.9 Hz, 1H, =CNH), 12.18 (s, 1H, CSNHCO), and 12.20 (s, 1H, CSNHCO); ^13^C (600 MHz, DMSO- d_6_): 94.4 (C), 119.5 (CH), 127.7 (CH), 141.4 (C), 141.7 (CH), 152.6 (C), 161.9 (CO), 164.2 (CO), and 178.4 (CS); mass spectrum: m/z 327.0218 [M + H]+ C_11_H_10_N_4_O_4_S_2_. Calculated: [M + H]+ 327.0217.

The rest of the compounds were obtained for screening for carbonic anhydrase inhibition from InterBioScreen (Chernogolovka, Moscow Region, Russia).

#### 3.1.2. Preparation of 1,3-Dicyclohexylbarbituric Acid **1b**

A solution of N,N-dicyclohexylcarbodiimide (1.97 g; 9.54 mmol) in anhydrous THF (35 mL) was added dropwise to a cold (0 °C) solution of malonic acid (0.489 g; 4.73 mmol) in anhydrous THF (35 mL) over a period of ∼30 min. The mixture was stirred and allowed to warm to room temperature over 2 h. (After 1 h, the mixture became very thick with precipitate, so further anhydrous THF (30 mL) was added to facilitate agitation.) The mixture was filtered and the filtrate evaporated to obtain a yellow solid, which was immediately slurried in ethanol (20 mL) and heated to reflux temperature. The mixture was then allowed to cool to room temperature, filtered, and the solid washed with cold ethanol (5 mL) to obtain the title compound (0.993 g, 72%) as a colorless solid. m.p. 200–203 °C.

#### 3.1.3. 4-{[(1,3-Dicyclohexyl-2,4,6-trioxotetrahydro-5(2H)-pyrimidinylidene)methyl]amino}benzenesulfonamide (**3**)

To a hot stirred solution of 1,3-dicyclohexylbarbituric acid **1b** (0.993 g; 3.4 mmol) in 1-butanol (25 mL) in the presence of slight excess of triethylorthoformate (0.532 g; 3.6 mmol), 4-aminobenzenesulfonamide (0.585 g; 3.4 mmol) was added, and the resulting reaction mixture was heated under reflux for 3 h. The solid formed was collected in hot state by suction filtration to obtain 4-{[(1,3-Dicyclohexyl-2,4,6-trioxotetrahydro-5(2H)-pyrimidinylidene)methyl]amino}benzenesulfonamide **3** (1.99 g; 93%), m.p. 273–274 °C. ^1^H (600 MHz, DMSO-d_6_): 1.23 (m, *J* = 5.0 Hz, 6H, 3CH_2_), 1.61 (dd, *J* = 5.0 Hz, 6 H, 3CH_2_), 1.80 (d, *J* = 5.0 Hz, 4H, 2CH_2_), 2.30 (q, *J* = 5.0 Hz, 4H, 2CH_2_), 4.66 (t, *J* = 5.0 Hz, 2H, 2NCH), 7.39 (s, 2H, SO_2_NH_2_), 7.73 (d, *J* =8.6 Hz, 2H, orto-ArH), 7.85 (2H, d, *J* =8.6 Hz, 2H, para-ArH), 8.66 (d, *J* = 13.9 Hz, 1H, CH=N), and 12.02 (d, *J* = 13.9 Hz, 1H, =CNH).

^13^C (600 MHz, DMSO- d_6_): 25.6, 26.6, 29.3 (cyclohexyl), 94.6 (C), 119.2 (CH), 127.8 (CH), 141.4 (C), 141.7 (CH), 151.0 (C), 152.6 (C). Mass spectrum: m/z 475.2009 [M + H]+ C_23_H_30_N_4_O_5_S. Calculated: [M + H]+ 475.2011.

### 3.2. Docking

Molecular modeling studies were conducted using AutoDock 4.2 software [31]. The Protein Data Bank (PDB) was utilized to retrieve the crystal structures of cytosolic isoforms hCA I (PDB code: 3W6H) and hCA II (PDB code: 3HS4), as well as hCA IX (PDB code: 3IAI) and hCA IV (PDB code: 1JD0) [32]. The entire procedure followed the methodology outlined in our previous research [33].

For ligand preparation, molecular structures were initially drawn using ChemDraw 12.0 [34]. Their geometry was then optimized with the MMFF94 molecular mechanics force field using LigandScout, where partial charges were calculated. Multiple conformations were generated for each ligand, and the most stable one was selected and saved as a mol2 file for conversion into pdbqt format via AutoDock Tools (ADT). Regarding protein preparation, PDB files were processed using SPDBV software, version 4.1 [35] to address any missing residues. Co-crystallized ligands and water molecules were removed, while polar hydrogens and charges were added using AutoDock Tools, version 4.2.6.

For docking simulations, the Lamarckian genetic algorithm was employed with default parameters for minimization. A total of 100 docking runs were performed, terminating after a maximum of 2,500,000 energy evaluations. All rotatable torsions were set as flexible, with a population size of 150. The translational step was defined at 0.2 Å, while quaternion and torsion steps were set to 5. Post-docking, the 100 docking solutions were clustered based on a root mean square deviation (RMSD) threshold of 1.0 Å, ranking the clusters by their lowest energy representatives. The highest-ranked binding mode was identified through AutoDock, depicting ligand interactions within the enzyme’s binding pocket. The resulting poses and binding interactions were visualized using LigandScout [36]. To validate the docking methodology, the original inhibitor of each enzyme was removed, re-docked, and compared with its initial position. The calculated RMSD values of 0.885 Å for hCA I, 0.966 Å for hCA II, 1.034 Å for hCA IX, and 1.127 Å for hCA IV confirmed the reliability of the protocol.

### 3.3. Drug-Likeness and Bioavailability Score

The ADMET properties and drug-likeness of all compounds were predicted using freely available computational tools. Specifically, the SwissADME (http://www.swissadme.ch) web server [37,38] was employed for ADMET analysis, while drug-likeness scores were assessed using Molsoft online (https://molsoft.com/about.html) [39]. Prior to prediction, all molecular structures were converted into their corresponding SMILES (Simplified Molecular Input Line Entry System) notation to ensure compatibility with the computational models.

### 3.4. Carbonic Anhydrase Inhibitory Activity

An Applied Photophysics stopped-flow instrument was used to assay the CA-catalyzed CO_2_ hydration activity [40]. Phenol red (at a concentration of 0.2 mM) was used as an indicator, working at the absorbance maximum of 557 nm, with 20 mM Hepes (pH 7.4) as a buffer and 20 mM Na_2_SO_4_ (to maintain constant ionic strength), following the initial rates of the CA-catalyzed CO_2_ hydration reaction for a period of 10–100 s. The CO_2_ concentrations ranged from 1.7 to 17 mM for the determination of the kinetic parameters and inhibition constants [41]. Enzyme concentrations ranged between 5 and 12 nM. For each inhibitor, at least six traces of the initial 5–10% of the reaction were used to determine the initial velocity. The uncatalyzed rates were determined in the same manner and subtracted from the total observed rates. Stock solutions of the inhibitor (0.1 mM) were prepared in distilled–deionized water, and dilutions up to 0.01 nM were made thereafter with the assay buffer. Inhibitor and enzyme solutions were preincubated together for 15 min at room temperature prior to the assay, to allow for the formation of the E–I complex. The inhibition constants were obtained by non-linear least-squares methods using PRISM 3 and the Cheng–Prusoff equation as reported earlier and represent the mean from at least three different determinations. All CA isoforms were recombinant proteins obtained in house, as reported previously [42].

## 4. Conclusions

In summary, nineteen newly synthesized p-aminobenzene-sulfonamide-based pyrimidine derivatives were assessed for their inhibitory effects on the cytosolic carbonic anhydrase isoforms hCA I, II, and IV, as well as the membrane-associated isoforms hCA IX and XII. The strongest inhibition was recorded for hCA II, where five molecules outperformed acetazolamide (AAZ). Among them, compound **14** exhibited the highest potency toward hCA II (Ki = 3.6 nM), whereas for hCA IV, compound **8** was the most effective (Ki = 5.4 nM), surpassing AAZ (Ki = 12.1 nM and 74 nM for hCA II and IV, respectively).

Conversely, compound **18** displayed activity against hCA IX (Ki = 32.4 nM) that was nearly comparable to AAZ (Ki = 25.8 nM). Although none of the derivatives exceeded the inhibitory power of AAZ against hCA IX or XII, several of them exhibited notable selectivity for hCA I, II, and IV. In particular, compound **2** demonstrated high selectivity indices (SI), calculated as 844.7 for hCA I, 109.1 for hCA II, and 228.8 for hCA IV.

To summarize, compounds **8** and **14** seem to be lead compounds for the development of more effective and safe inhibitors against hCA II and IV and hCA II, respectively.

## Figures and Tables

**Table 1:** Inhibitory activity of compounds against hCA I, hCA II, hCA IV, hCA IX, and hCA XII.

K_I_ (nM)*					
Cmp	hCA I	hCAII	hCA IV	hCAIX	hCAXII
**2**	8030 ± 787	926.9 ± 91.5	1945 ± 170	110.3 ± 9.0	8.5 ± 0.5
**3**	8604 ± 438	516.9 ± 31.3	2028 ± 139	41.0 ± 3.2	31.1 ± 2.5
**4**	65.7 ± 5.2	76.8 ± 5.2	4520 ± 230	97.2 ± 4.8	58.1 ± 5.0
**5**	550.8 ± 52.5	87.5 ± 6.4	6893 ± 511	142.5 ± 11.7	91.4 ± 6.6
**6**	268.0 ± 26.5	92.5 ± 4.8	1285 ± 112	34.8 ± 2.0	23.7 ± 1.4
**7**	366.4 ± 25.4	7.0 ± 0.5	28.5 ± 1.8	35.6 ± 2.1	9.5 ± 0.6
**8**	78.7 ± 4.7	6.1 ± 0.5	5.4 ± 0.3	39.1 ± 3.2	8.7 ± 0.5
**9**	394.8 ± 23.9	24.4 ± 1.5	320.2 ± 28.2	30.3 ± 2.1	7.5 ± 0.5
**10**	1301 ± 92	6.9 ± 0.5	1907 ± 162	38.2 ± 3.1	63.5 ± 4.3
**11**	783.9 ± 44.2	26.5 ± 2.2	1649 ±102	75.3 ± 4.3	8.6 ± 0.4
**12**	8090 ± 625	92.2 ± 5.0	2304 ± 157	96.6 ± 6.4	89.0 ± 6.4
**13**	349.5 ± 30.7	8.3 ± 0.6	635.7 ± 48.8	112.2 ± 7.8	56.7 ± 5.0
**14**	266.5 ± 19.4	3.6 ± 0.2	29.5 ± 2.6	43.1 ± 2.4	75.1 ± 4.7
**15**	1794 ± 135.3	72.3 ± 6.9	1762 ± 101	123.0 ± 9.0	9.7 ± 0.7
**16**	2132 ± 149	92.7 ± 8.3	1458 ± 93	46.4 ± 3.2	72.9 ± 6.4
**17**	416.6 ± 24.8	80.8 ± 7.2	185.1 ± 11.8	43.0 ± 3.3	70.5 ± 3.6
**18**	1671 ± 144	426.7 ± 31.2	720.5 ± 40.4	32.4 ± 1.8	21.5 ± 1.2
**19**	1738 ± 151	698.1 ±41.9	2258 ± 132	43.9 ± 3.2	67.3 ± 3.8
**20**	403.5 ± 37.4	76.7 ± 5.9	2148 ± 169	162.2 ± 8.9	9.1 ± 0.6
**AAZ**	250.0 ± 15.6	12.1 ± 0.7	74.0 ± 5.2	25.8 ± 1.3	5.7 ± 0.5

**Table 2:** Selectivity indices for the inhibition of transmembrane hCA IX and XII isoforms over cytosolic hCA I, II and IV for the synthesized compounds.

Compound	I/IX	II/IX	IV/IX	I/XII	II/XII	IV/XII
**2**	72	8.40	17.63	944.71	109.1	228.82
**3**	209	12.61	49.46	276.66	16.62	65.21
**4**	0.68	0.79	46.50	1.13	1.32	77.80
**5**	3.87	0.61	48.37	6.03	0.96	75.42
**6**	7.70	2.66	36.93	11.31	3.90	54.22
**7**	10.29	0.002	0.80	38.57	0.74	3.0
**8**	2.01	0.16	0.14	9.05	0.70	0.62
**9**	13.03	0.85	10.57	52.64	2.53	42.69
**10**	34.06	0.18	49.21	20.49	0.109	30.03
**11**	10.41	0.35	21.90	91.15	3.08	191.74
**12**	83.75	0.96	23.85	90.90	1.04	25.89
**13**	3.13	0.07	5.67	6.16	0.15	11.21
**14**	6.18	0.08	0.68	3.55	0.05	0.39
**15**	14.59	0.59	14.35	184.95	7.45	18.14
**16**	45.95	1.997	31.42	29.25	1.27	20
**17**	9.69	1.88	4.21	5.91	1.15	2.63
**18**	51.57	13.17	22.24	77.72	19.85	33.51
**19**	39.59	15.90	51.44	25.82	10.37	33.55
**20**	2.49	0.48	13.24	44.34	8.43	236.0
**AAZ**	9.69	0.47	2.87	43.86	2.12	12.98

**Table 3:** Molecular docking free binding energies (kcal/mol) and interactions of selected compounds on hCA I, II, IV and IX isoforms.

No.	*h*CA Isoform	Estimated Free Binding Energy (Kcal/mol)	Chelating the Zn (II) Ion	Residues Involved in H-Bond Interactions	Residues Involved in Hydrophobic Interactions
**2**	hCA I	−5.13	No	-	Val121
	hCA II	−6.39	No	-	Ile60, Val62
	hCA IX	−6.14	No	-	Leu198, Thr200
	hCA IV	−5.57	No	-	Val121, Leu198
**4**	hCA I	−9.53	Yes	His200 (N···H, 2.57 Å), Thr199 (O···H, 2.86 Å)	Ile60, Ile198
	hCA II	−7.02	Yes	His200 (O···H, 2.84 Å)	Ile60, Val62
	hCA IX	−6.25	No	-	Leu198, Thr200
	hCA IV	−4.03	No	-	-
**7**	hCA I	−4.69	No	-	Leu198
	hCA II	−9.18	Yes	His200 (O···H, 1.94 Å)	Ile60, Val62, Ala132
	hCA IX	−8.16	Yes	Thr199 (N···H, 2.97 Å)	Leu198, Thr200
	hCA IV	−8.10	Yes	Thr199 (O···H, 2.78 Å)	Ile141, Leu198
**8**	hCA I	−10.35	Yes	His200 (O···H, 2.54 Å), Thr199 (O···H, 2.73 Å)	Val62, Ile60, Ile198
	hCA II	−10.11	Yes	His94 (N···H, 2.58 Å), Thr199 (O···H, 3.35 Å)	Thr204
	hCA IX	−8.98	Yes	His94 (N···H, 2.58 Å), Thr199 (O···H, 3.11 Å), His96 (N···H, 2.63 Å)	Trp5, Val121, Leu198
	hCA IV	−11.86	Yes	Ser197 (N···H, 2.74 Å), Thr199 (O···H, 3.43 Å), Cys203 (O···H, 2.71 Å)	Leu141, Val121, Thr200, Leu198
**10**	hCA I	−5.24	No	-	Val121, Leu198, Thr200
	hCA II	−9.13	Yes	His94 (O···H, 1.75 Å)	Val121, Phe131, Leu198
	hCA IX	−6.02	No	-	Leu198, Thr200
	hCA IV	−4.93	No	-	Val121, Leu198
**12**	hCA I	−6.28	Νο	-	Ile60, Ile198
	hCA II	−6.85	Yes	-	Ile60, Val62
	hCA IX	−4.69	No	-	Thr200
	hCA IV	−5.91	No	-	Leu198
**14**	hCA I	−6.58	No	-	Val121, Leu198
	hCA II	−9.74	Yes	Thr199 (N···H, 1.83 Å)	Trp5, Val121, Phe131, Thr200
	hCA IX	−7.63	No	Thr200 (O···H, 2.35 Å)	Val131, Leu198, Thr200
	hCA IV	−8.20	Yes	Thr199 (O···H, 2.74 Å)	Ile141, Leu198, Thr200
**AAZ**	hCA I	−7.28	No	Gln92, Thr199	Leu198, Pro201
	hCA II	−8.87	Yes	Thr199, Thr200	Val121, Phe131, Leu198, Trp209
	hCA IX	−8.46	Yes	Thr199, Thr200	Val121, Val143, Val131, Leu198, Trp209
	hCA IV	−7.15	No	Thr199, Thr200	Leu198

**Table 4:** Drug-likeness and bioavailability score of tested compounds.

Property		Predicted Value							Unit
	1	2	3	4	5	6	7	8	
**Molecular weight**	326.35	474.57	320.37	392.39	455.33	355.39	310.29	327.34	Numeric (g/mol)
**Num. of rotatable bonds**	3	5	5	5	4	6	3	3	Numeric
**Num. of H-bond acceptors**	5	6	6	7	5	6	7	7	Numeric
**Num. of H-bond donors**	4	2	4	2	2	4	4	4	Numeric
**TPSA**	170.86	138.26	12.23	159.46	106.35	194.71	177.48	186.71	Numeric (Å^2^)
**Consensus Log *P*_o/w_**	−0.38	2.17	0.79	−0.43	3.71	−0.22	−1.25	−0.13	Average of five methods
**Solubility**	Very	Mod	Mod	Very	Poor	Very	Very	Soluble	Categorical
**GI absorption**	Low	Low	Low	Low	Low	Low	Low	Low	Categorical
**P-gp substrate**	No	Yes	No	No	No	No	No	No	Categorical
**CYP2D6 inhibitor**	No	No	No	No	Yes	No	No	No	Categorical
**CYP3A4 inhibitor**	No	Yes	No	No	Yes	No	No	No	Categorical
**Lipinski**	0	0	0	1	0	0	0	0	Categorical
**Bioavailability score**	0.55	0.55	0.55	0.55	0.55	0.55	0.55	0.55	
**PAINS**	1 *	1	0	0	0	0	0	0	Categorical
**Drug-likeness score**	0.29	0.27	0.56	0.81	0.17	0.94	0.83	1.10	
**-**	9	10	11	12	13	14	15	16	
**Molecular weight**	311.27	382.46	428.46	372.40	358.37	418.42	459.91	443.45	Numeric (g/mol)
**Num. of rotatable bonds**	3	8	4	6	5	7	5	5	Numeric
**Num. of H-bond acceptors**	9	7	6	6	6	8	6	7	Numeric
**Num. of H-bond donors**	4	3	2	2	2	2	3	3	Numeric
**TPSA**	175.18	168.95	130.42	132.53	132.53	150.99	144.56	144.56	Numeric (Å^2^)
**Consensus Log *P*_o/w_**	−0.53	1.41	0.75	0.84	0.59	0.61	1.67	1.58	Average of five methods
**Solubility**	Soluble	Mod	Mod	Mod	Mod	Mod	Poor	Poor	Categorical
**GI absorption**	Low	Low	High	High	High	Low	Low	Low	Categorical
**P-gp substrate**	No	No	Yes	No	No	Yes	Yes	YES	Categorical
**CYP2D6 inhibitor**	No	No	No	No	No	No	No	No	Categorical
**CYP3A4 inhibitor**	No	No	No	No	No	No	No	No	Categorical
**Lipinski**	0	0	0	0	0	0	0	0	Categorical
**Bioavailability score**	0.55	0.55	0.55	0.55	0.55	0.55	0.55	0.55	
**PAINS**	0	0	0	0	0	0	0	0	Categorical
**Drug-likeness score**	0.42	0.76	0.84	0.63	1.13	0.95	1.47	1.33	

Consensus Log P_o/w_: consensus logarithm of partition coefficient (octanol/water) predicted as an average of five methods (iLOGP, XLOGP3, WLOGP, MLOGP, and SILICOS-IT); TPSA: topological polar surface area (Å). * 1 alert: ene_six_het_A.

## Data Availability

The original contributions presented in this study are included in the article and Supplementary Materials. Further inquiries can be directed to the corresponding author.

## Supplementary Materials

The following supporting information can be downloaded at: https://www.mdpi.com/article/10.3390/ijms27062725/s1. References [43,44,45,46,47,48,49] are cited in the Supplementary Materials.
