# Supplementary material for: p-Aminobenzene-Sulfonamide Derivatives of Substituted Pyrimidines as Human Carbonic Anhydrase Inhibitors
Source: Int J Mol Sci. 2026 Mar 17;27(6):2725. doi: 10.3390/ijms27062725 (PMC13026987; doi:10.3390/ijms27062725)
Supplement: Supplementary file 1 [file ijms-27-02725-s001.zip › ijms-4187656-supplementary.pdf]

p-Aminobenzene-sulfonamide derivatives of Substituted Pyrimidines as human Carbonic Anhydrase Inhibitors.

Andrea Angeli,[1]. Anthi Petrou,[2] Kartsev V.,[3] Prezent M.,[4] Samvel Sirakanyan, [5]Athina Geronikaki,[2] Claudiu T. Supuran [1]

[1]Neuro Farba Department, Sezione di Scienze Farmaceutiche, Università degli Studi di Firenze, Via Ugo Schiff 6, 50019 Sesto Fiorentino (Florence), Italy; claudiu.supuran@unifi.it
[2]School of Health, Department of Pharmacy, Aristotle University of Thessaloniki, 54124, Greece
[3]InterBioscreen, Moscow, Russia
[4]Zelinsky Institute of Organic Chemistry, Leninsky prospect 11991,Moscow, Russia,pre1962@mail.ru

[5] Scientific Technological Center of Organic and Pharmaceutical Chemistry of National Academy of Science of Republic of Armenia Institute of Fine Organic Chemistry of A. L. Mnjoyan, Armenia 0014, Yerevan, Ave. Azatutyan 26 E-mail: shnnr@mail.ru

Table of contents

1.Schemes of synthesis of some compounds

2.^{1}H-NMR spectra of compounds **2-20**

3.^{13}C-NMR of compounds **2,3**

All commercially available chemicals were of analytical quality and solvents were either of analytical quality or used after further purification. The reactions were monitored by thin-layer chromatography using a Merck type silica gel 60 GF 254 thin-layer plate, with the developed chromatograms visualised under 254 nm UV light.

Melting point determinations were performed using a Büchi M-560 melting point apparatus and data are given in ^{o}C.

LS/MS measurements were performed on a Agilent Technologies 1260 infinity with Agilent 6230 TOF LC/ mass spectrometer.

Nuclear magnetic resonance (NMR) measurements were carried out using a Varian MercuryPlus Bruker Avance III (^{1}H: 600 MHz, ^{13}C: 150MHz) spectrometer equipped with a standard and a cryogenic head, respectively. For structural assignment, samples were prepared by dissolving 10 mg of the solid compounds in 600 μL of DMSO-d6. The NMR spectra were recorded at room temperature using the 2H signal of the solvent as lock and tetrame-thylsilane as internal standard (TMS = 0 ppm) or spectra were referenced to the solvent signal (^{1}H: 2.50 ppm and ^{13}C: 39.50 ppm). The solvent is given in the characterization of the compound. Chemical shift values (δ) are in ppm and coupling constants (J) in Hz. The multiplicity is given using designations commonly used in spectroscopy. Structural characterization was performed using ^{1}H, ^{13}C experiments.

1.

On condensing aminopyrazoles with maleic anhydride in acetic acid on heating to 80-100 °C , the compounds **4-8** were synthesized. [41]

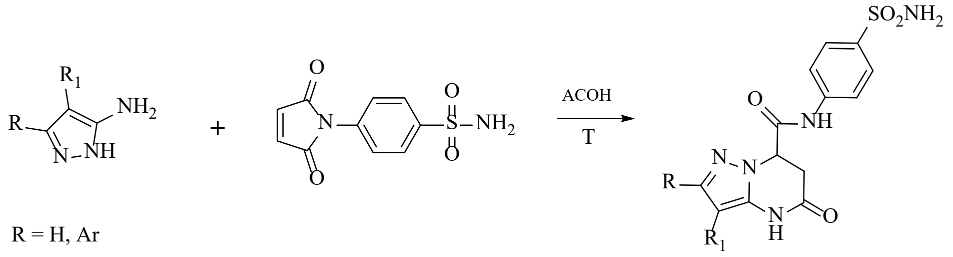

4-8

For the synthesis of compounds **9,14,16,** chloroacetamide and chloropropionylamide were probably used from 4-aminobenzenesulfamide.[42].

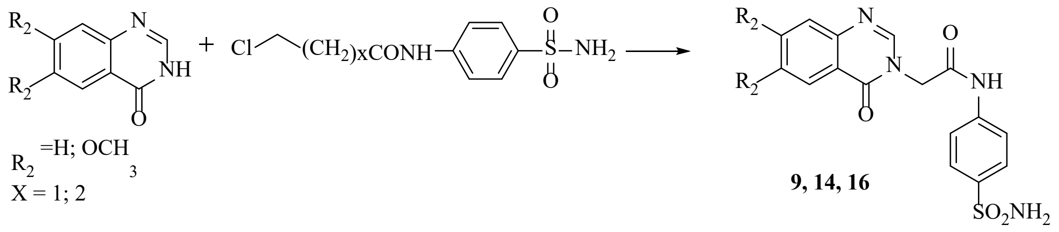

9, 14, 16

To obtain compounds **10-12**, diazotization of 1,3-pyrimidinediones with aryldiazonium salt of 4-aminobenzenesulfamide was apparently used. [43]

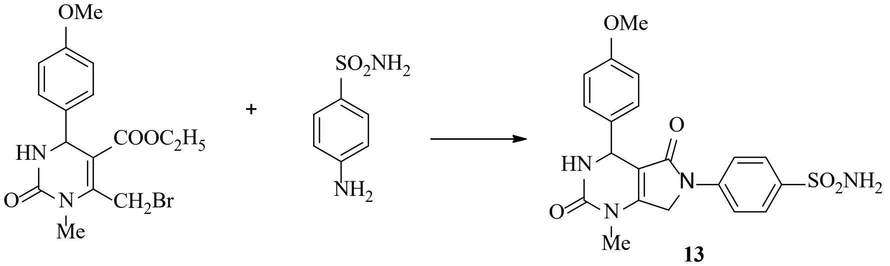

13

Compound 13 was synthesized according to [44]

Compound **15** was apparently synthesized by alkylation of theophylline with the corresponding chloroacetamide .

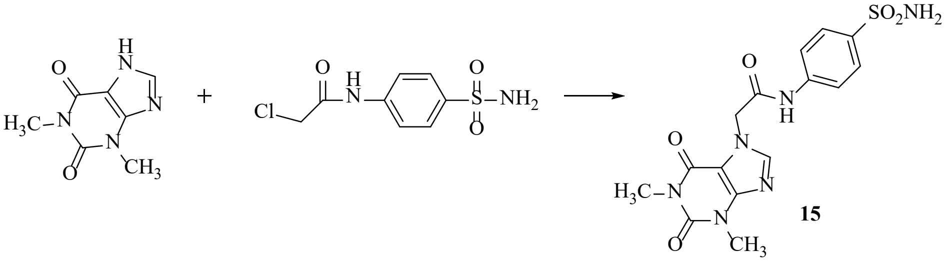

15

Pyrimidines **17-18** were apparently prepared by alkylation of the corresponding 2-mercaptopyrimidines with chloroacetamide from 4-aminobenzenesulfamide. [45]

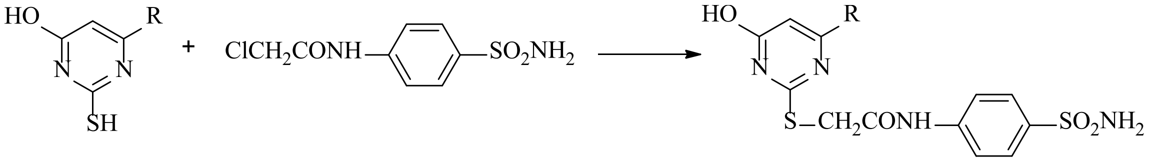

R = C_3H_7; NH_2

17, 18

Compound **19** is apparently obtained by reaction of 2-benzoylaniline and isothiocyanate obtained from 4-aminobenzenesulfamide. [46]

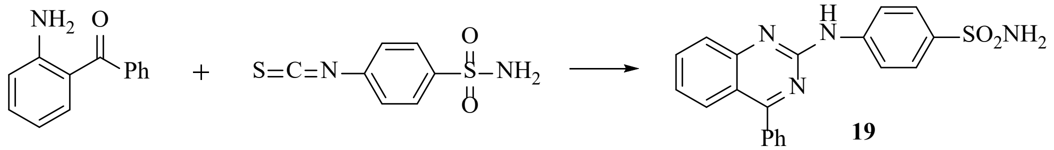

19

Guanidine 20 was apparently obtained from the corresponding cyanamide and 4-aminobenzenesulfamide [47].

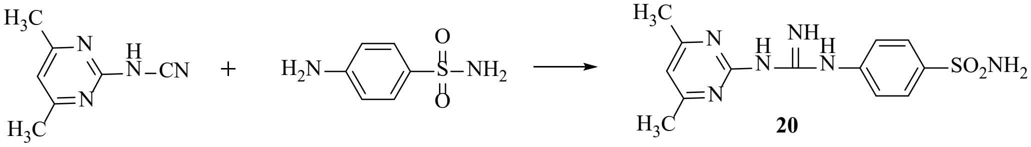

20

References

43. Filimonov , S.I: Korsakov,M.K.; Chirkova ,Zh.V.;Abramov , I.G.; Stashina ,G.A.; Firgang ,S.I.; Kovygin , Yu.A.; Shikhaliev , Kh.S.CONDENSATION OF 5-AMINO-4-ARYLPYRAZOLES WITH ITACONIC ACID AND MALEIC ANHYDRIDE. Chem.Heterocyc. Comp/ds.,**2013**, *49*, 7 (Russian Original Vol. 49, No. 7, July, 2013.

44.Mazur, A. I.; Sinyak, R. S.; Kovalenko, S. I.; Belenichev, I. F.; Muzilev, V. V.; et al Synthesis and properties of α(or β)-(3,4-dihydro-4-oxoquinazolin-3-yl)carboxylic acids and their derivatives By: *Ukrainskii Khimicheskii Zhurnal* (Russian Edition**)** **1995**, *61(7-8)*, 54-57 .

45. Nesynov, E. P.; Besprozvannaya, M. M Arylation of monothiobarbituric acid by aryldiazonium salts . *Khimiya Geterotsiklicheskikh Soedinenii* **1971**, *7(9)*, 1271-1275.

46.Zigeuner, G.; Knopp, Ch. Heterocycles. XXII. 3,4,6,7-Tetrahydro-5H-pyrrolo[3,4d]pyrimidine-2(1H)-5-diones.*Monatsh. Chem.* **1970**, *101*(5), 1541-1546..

47. Scotese, A.C.; Santilli,A.A.; Nelson, G.L..Synthesis and antiarrhythmic activity of substituted (2-pyrimidinylthio)acetamidoximes . *J Med Chem.* **1975,** *18(8)*,852-854.

48. Jatangi, Nagesh; Palakodety, Radha Krishna I2-Catalyzed oxidative synthesis of N,4-disubstituted quinazolines and quinazoline oxides.Org. & Biomol. Chem.**2019**, *17*(15), 3714-3717

49.Kryl'skii, D. V.; Shikhaliev, Kh. S.; Shestakov, A. S.; Liberman, M. Arylbiguanides in heterocyclization reactions M. *Russian Journal of General Chemistry* **2005,**, *75*(2), 303-310

2.^{1}H-NMR spectra of compounds 2-20

Compound **2**

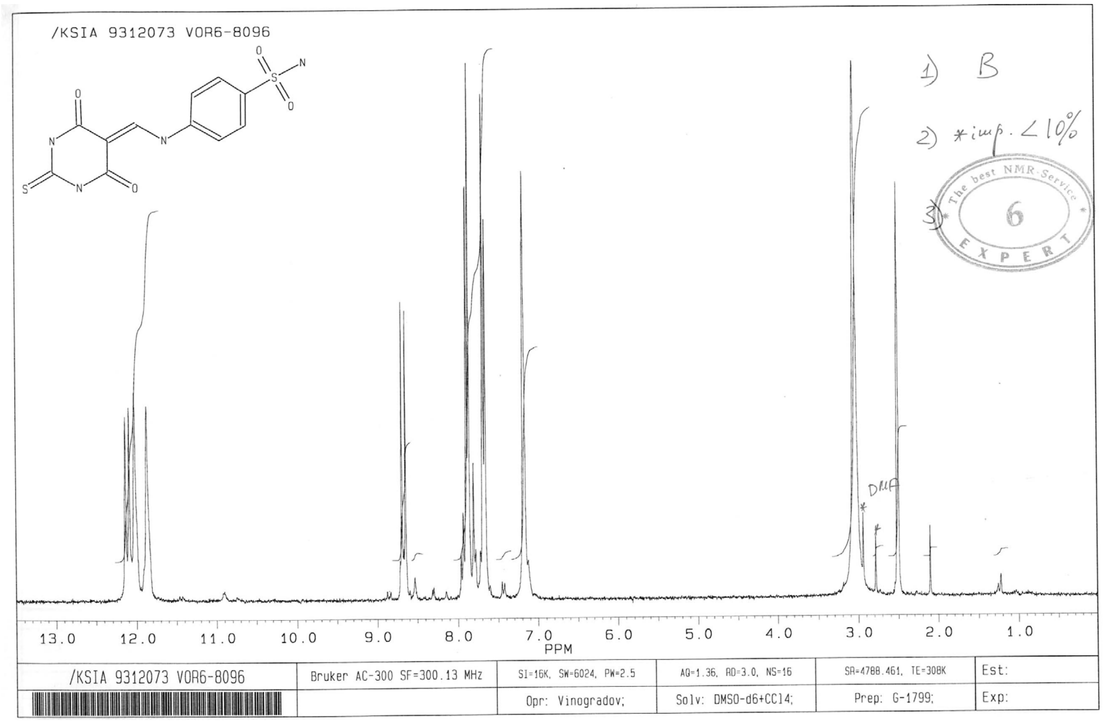

Compound **3**

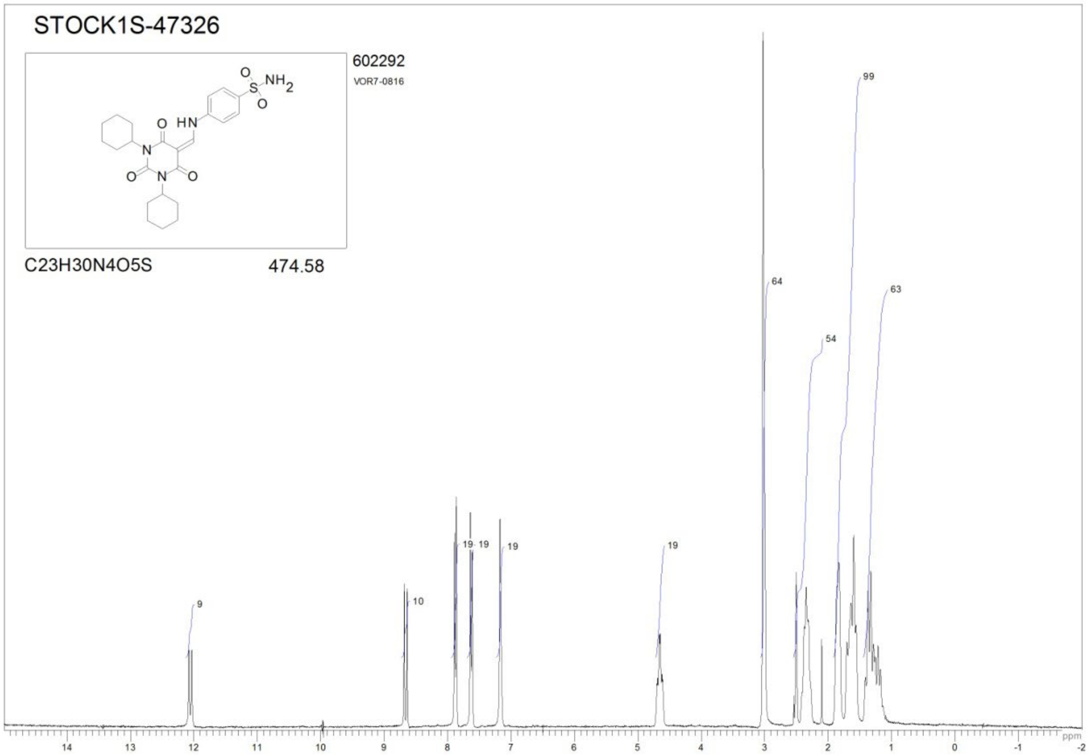

Compound **4**

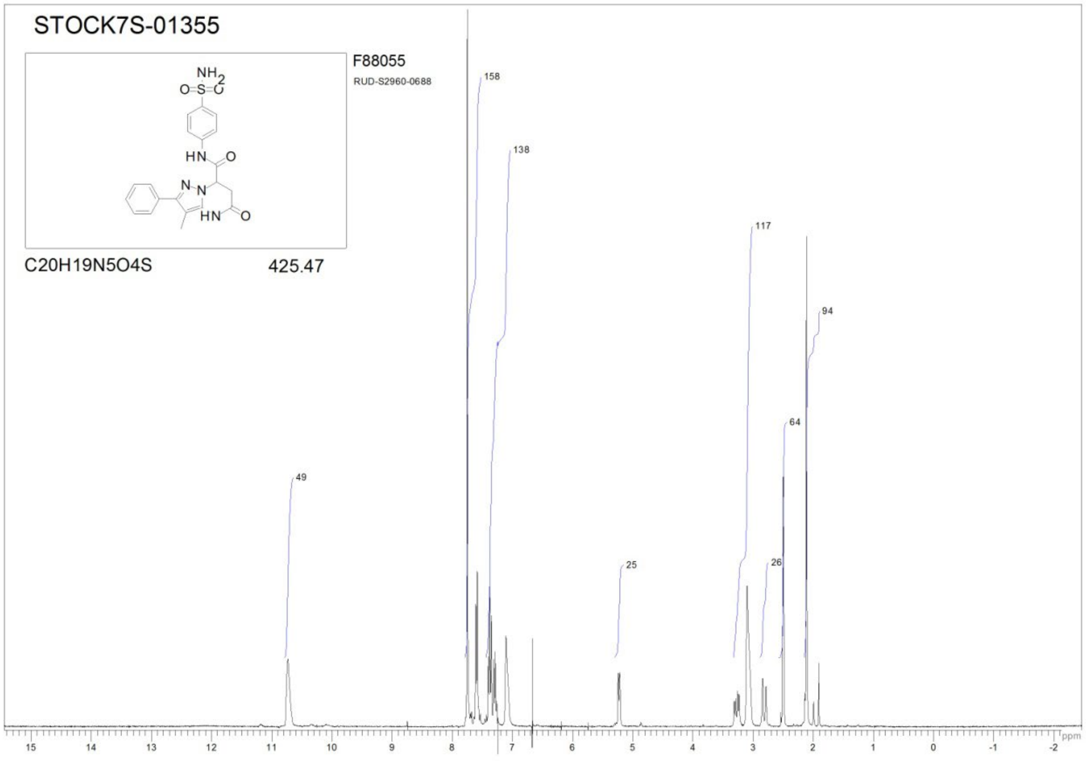
STOCK7S-01355
F88055
RUD-S2960-0688
C20H19N5O4S
425.47
ppm

Compound **5**

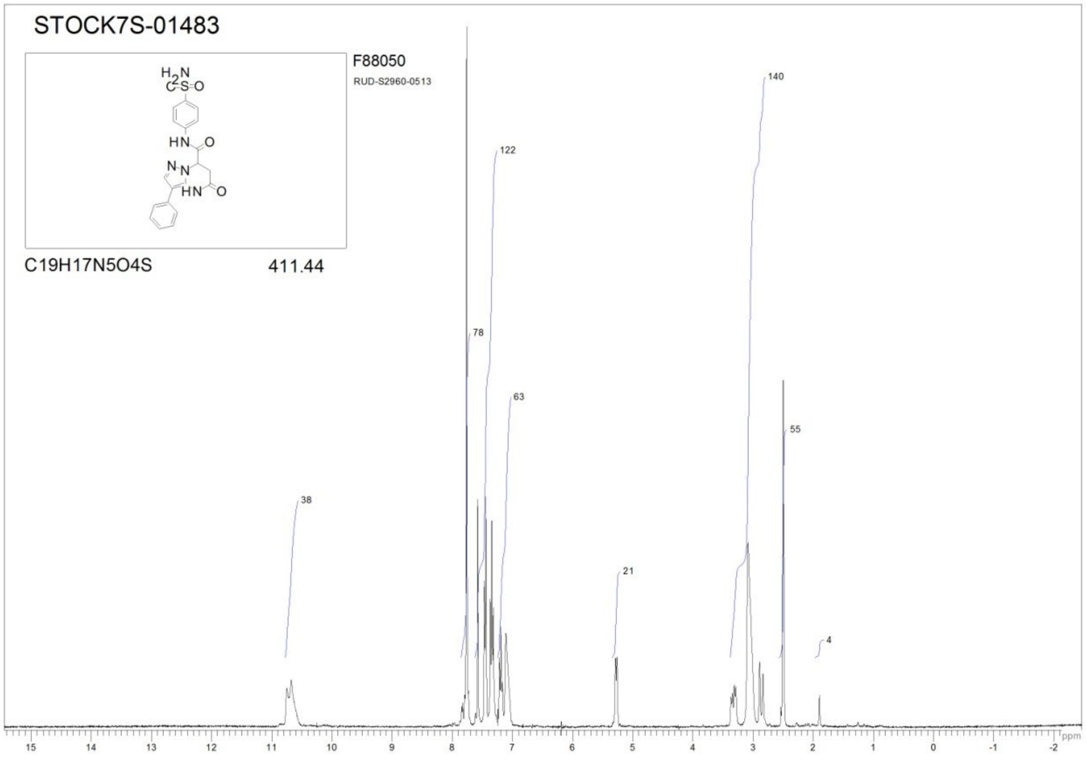
STOCK7S-01483
F88050
RUD-S2960-0513
C19H17N5O4S
411.44
ppm

Compound **6**

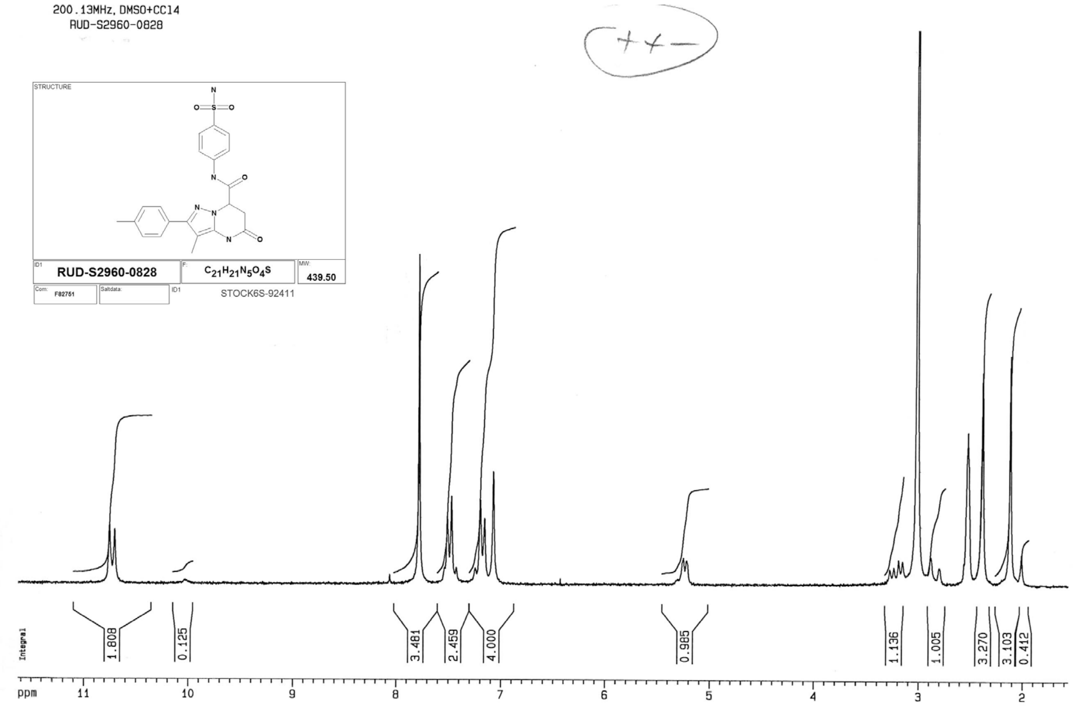

Compound **7**

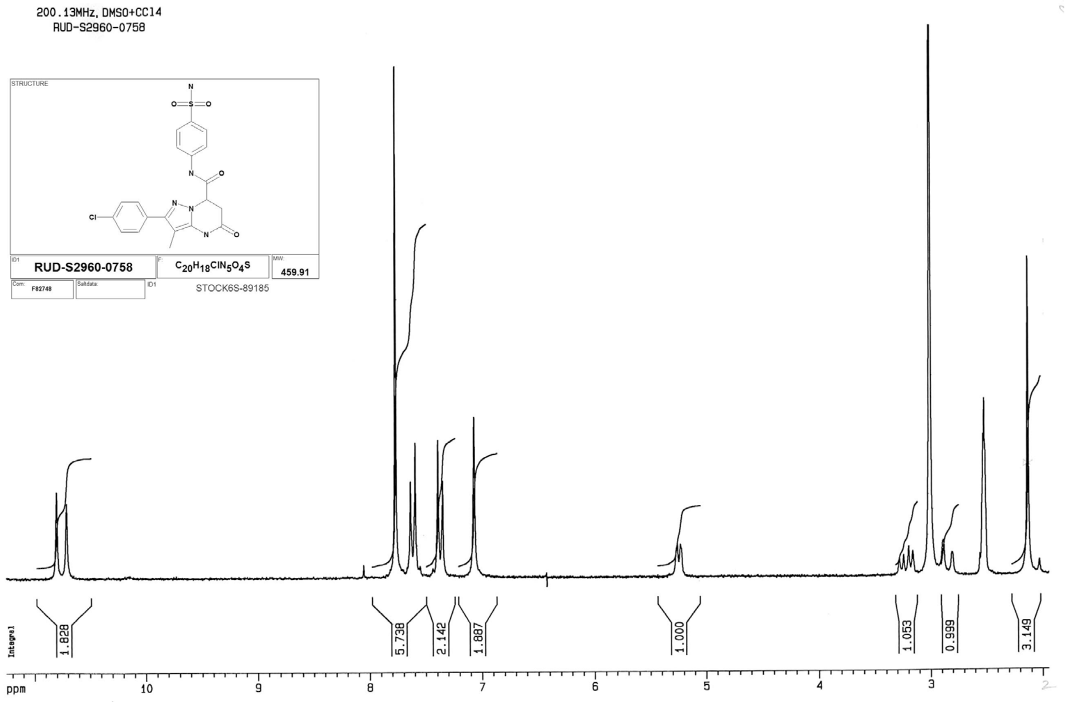

Compound 8

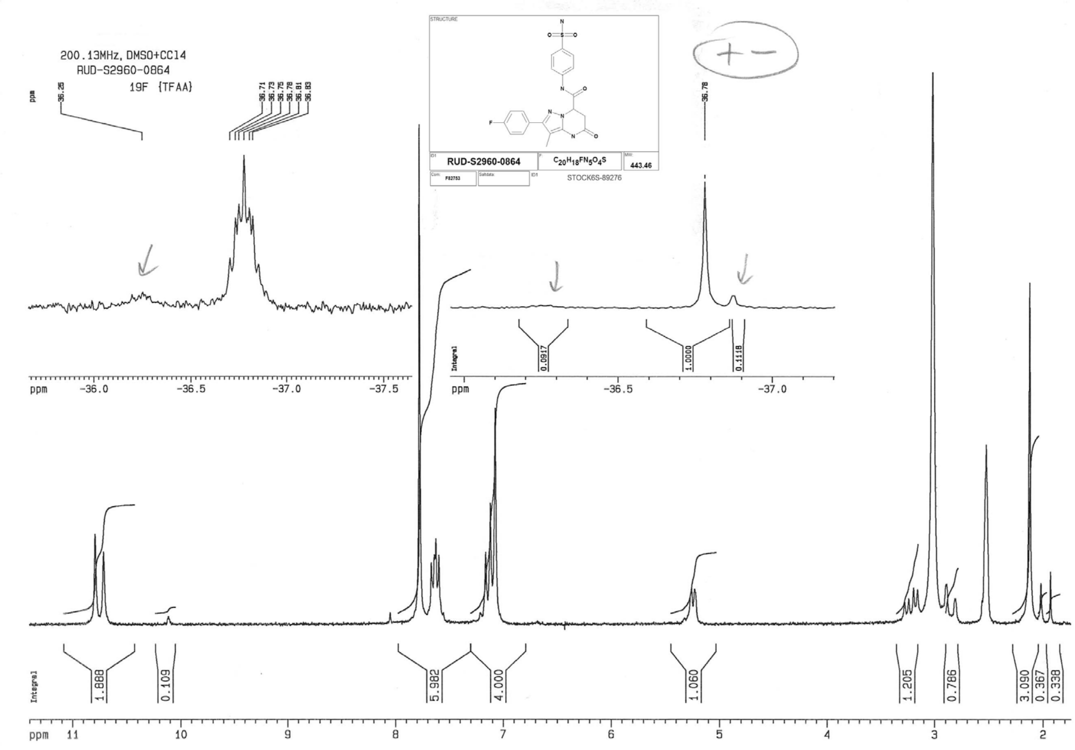

Compound **9**

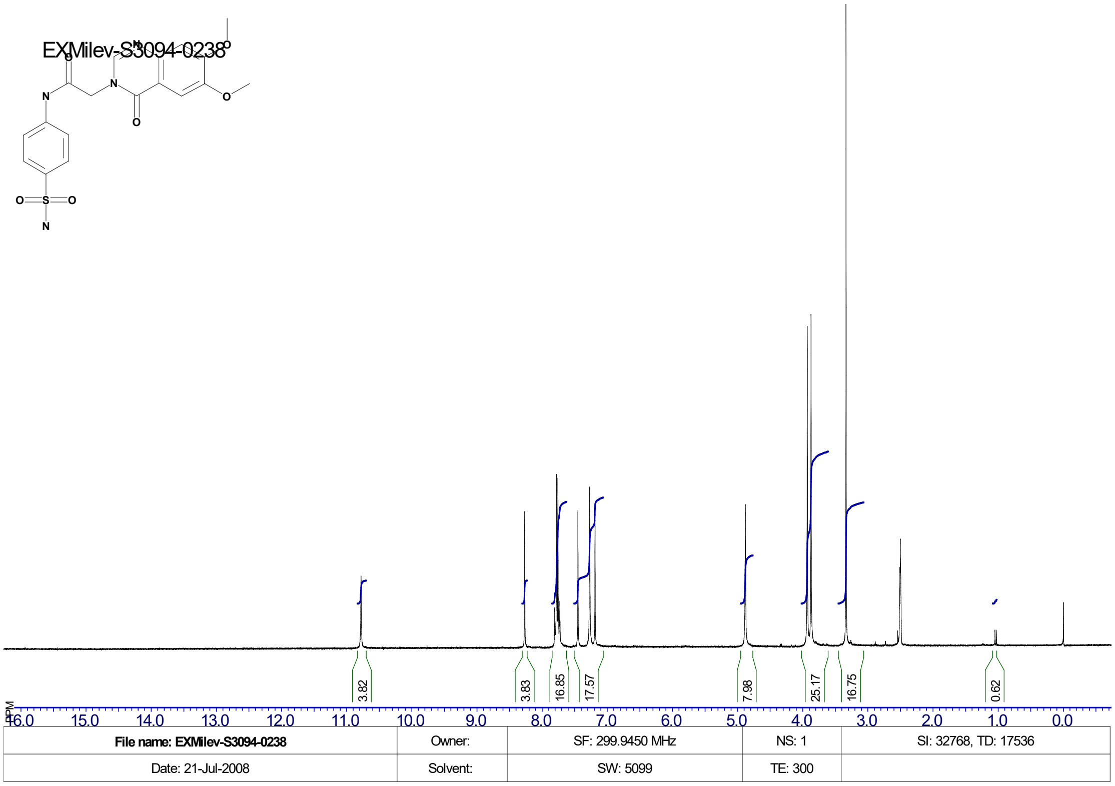
EXMilev-S3094-0238
File name: EXMilev-S3094-0238
Owner:
SF: 299.9450 MHz
NS: 1
SI: 32768, TD: 17536
Date: 21-Jul-2008
Solvent:
SW: 5099
TE: 300
3.82
3.83
16.85
17.57
7.98
25.17
16.75
0.62
PPM

Compound **10**

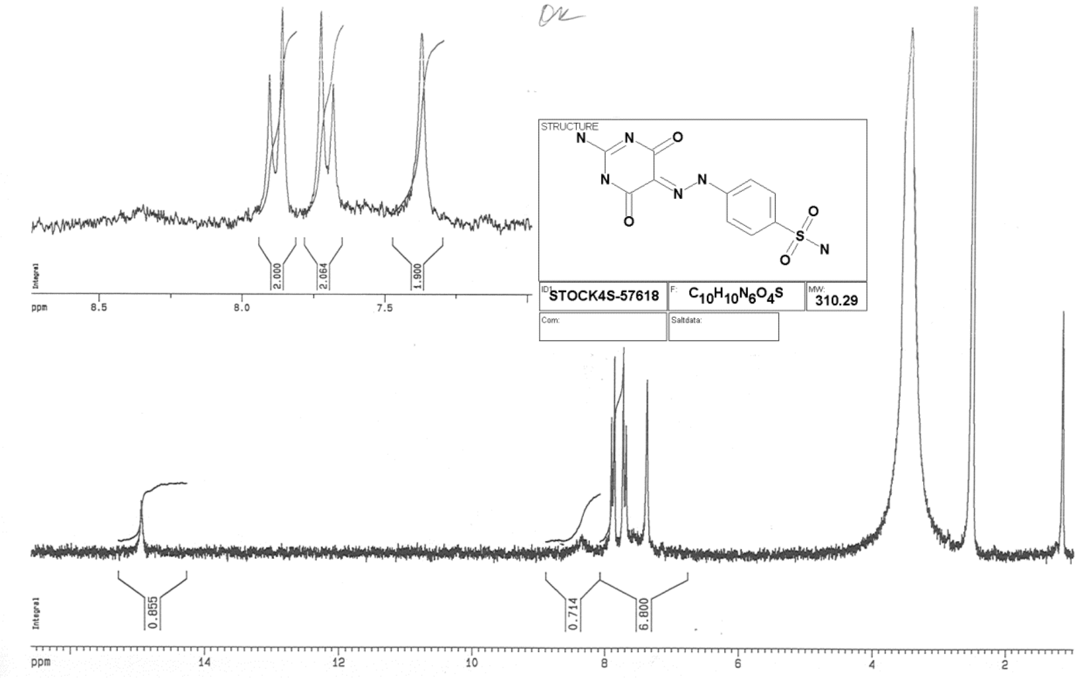
STRUCTURE
STOCK4S-57618
$C_{10}H_{10}N_6O_4S$
310.29
Com:
Saltdata:
2.000
2.064
1.900
0.855
0.714
6.800
Integral
ppm

Compound **11**

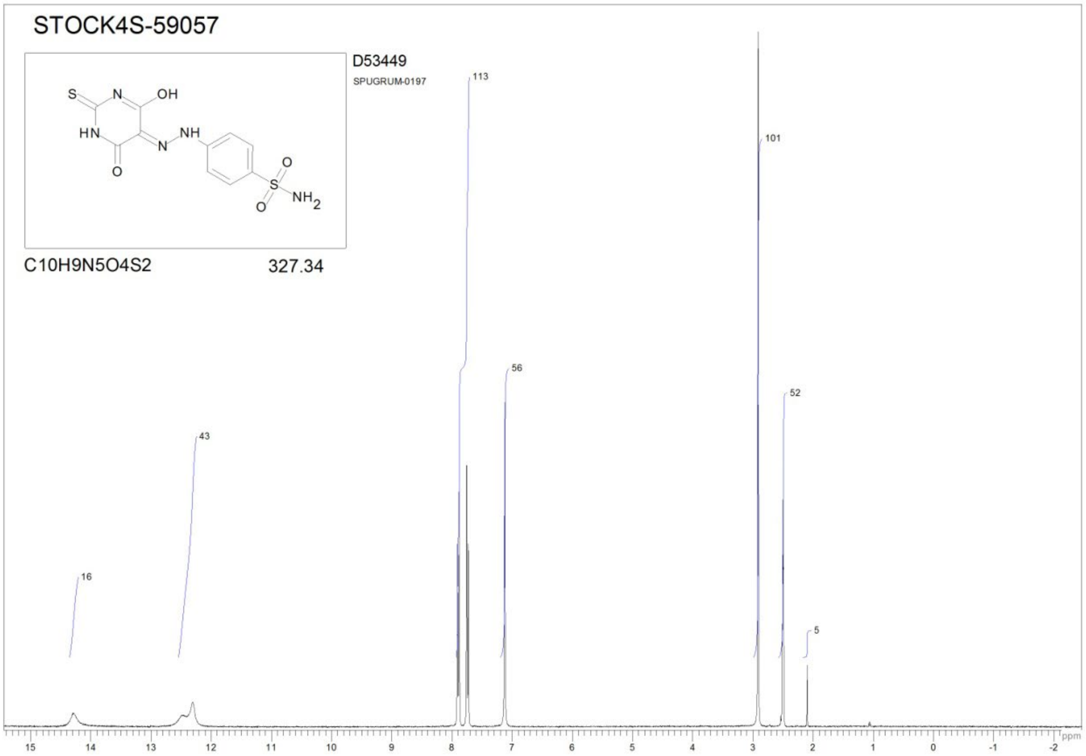

Compound **12**

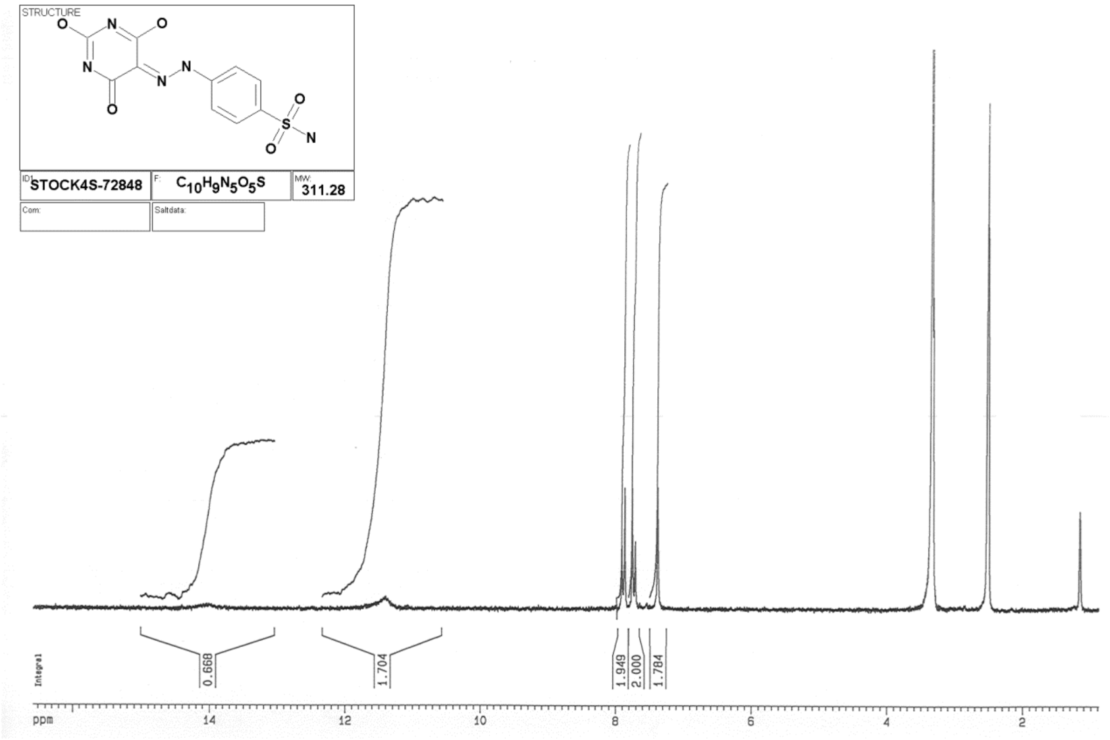
STRUCTURE
STOCK4S-72848
$C_{10}H_9N_5O_5S$
311.28
Com:
Saltdata:
Integral
0.668
1.704
1.949
2.000
1.784
ppm

Compound **13**

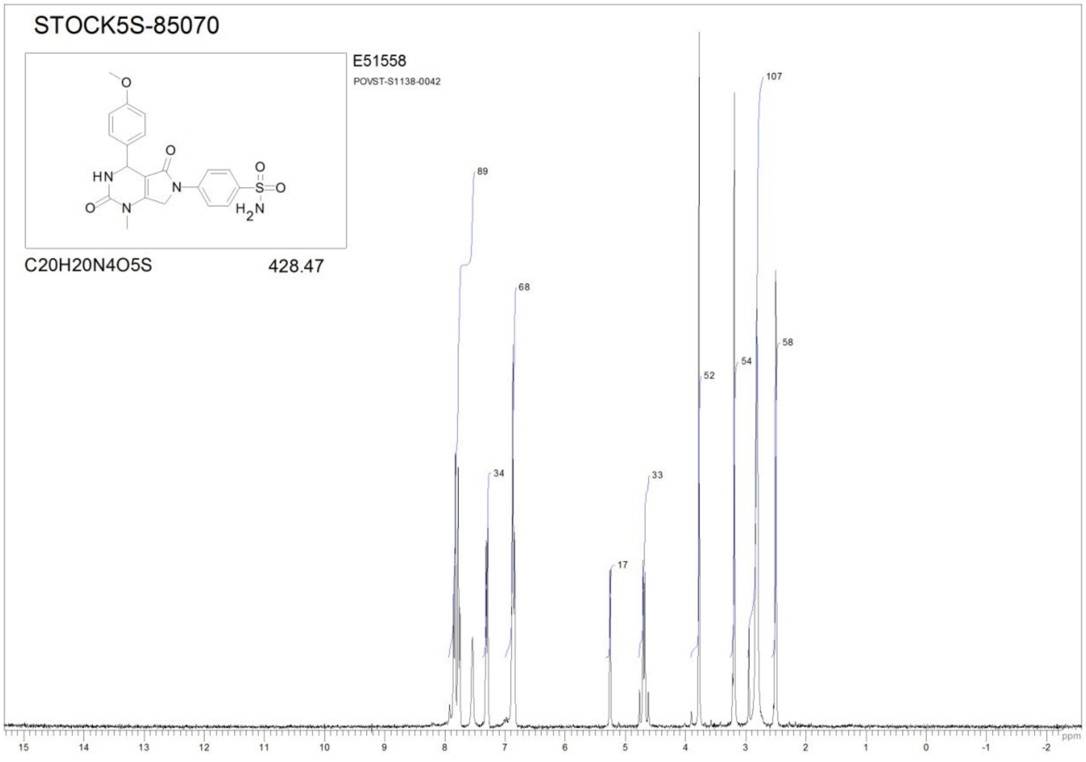
STOCK5S-85070
E51558
POVST-S1138-0042
C20H20N4O5S
428.47
89
68
34
17
33
52
54
107
58
ppm

Compound **14**

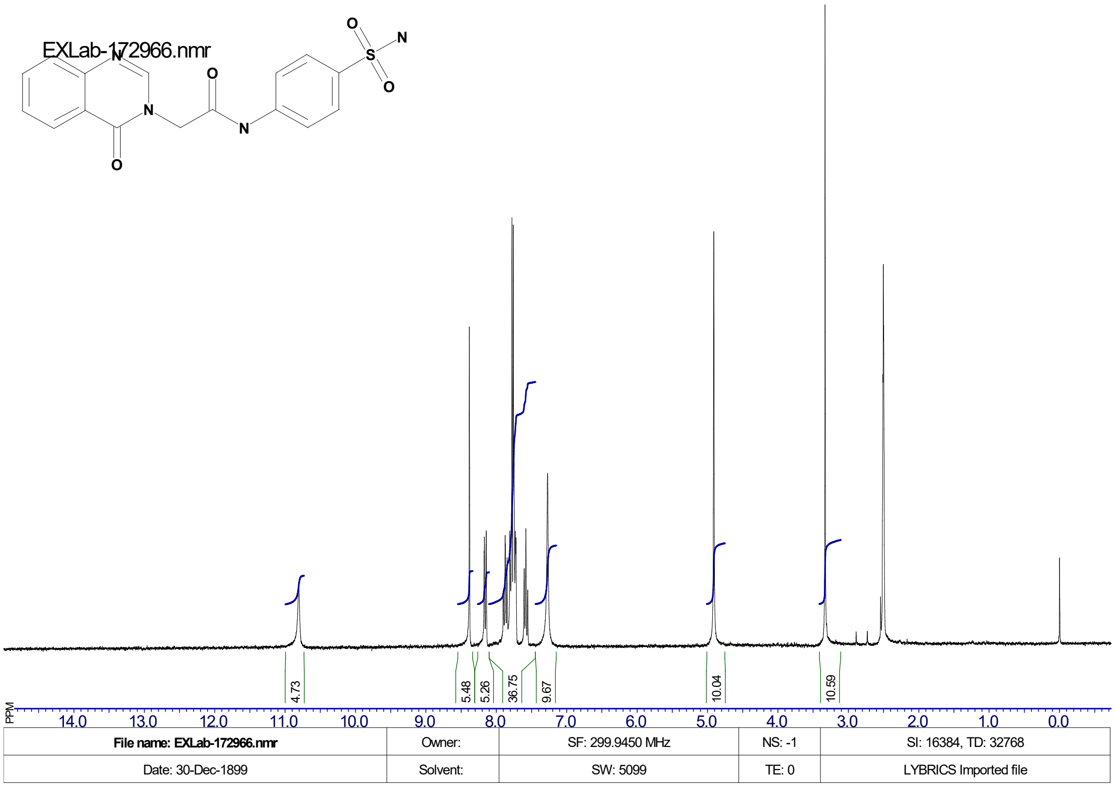

Compound **15**

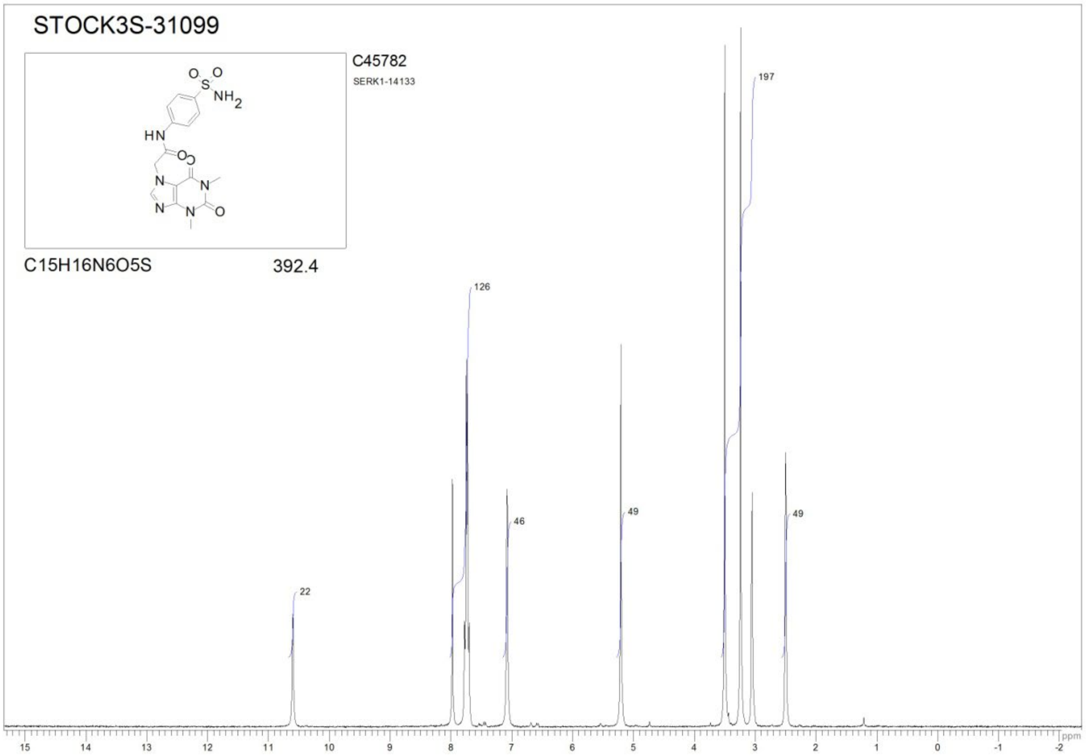
STOCK3S-31099
C45782
SERK1-14133
C15H16N6O5S
392.4
197
126
22
46
49
49
ppm

Compound **16**

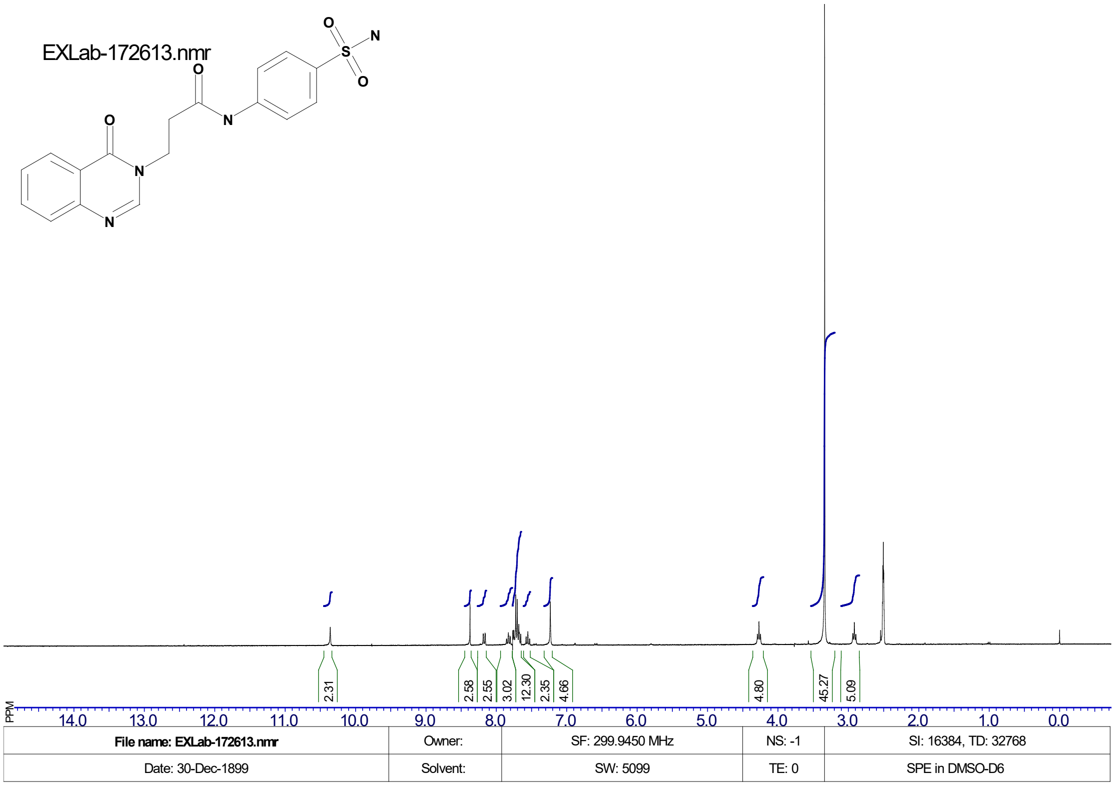
EXLab-172613.nmr
2.31
2.58
2.55
3.02
12.30
2.35
4.66
4.80
45.27
5.09
PPM
14.0 13.0 12.0 11.0 10.0 9.0 8.0 7.0 6.0 5.0 4.0 3.0 2.0 1.0 0.0
File name: EXLab-172613.nmr
Owner:
SF: 299.9450 MHz
NS: -1
SI: 16384, TD: 32768
Date: 30-Dec-1899
Solvent:
SW: 5099
TE: 0
SPE in DMSO-D6

Compound **17**

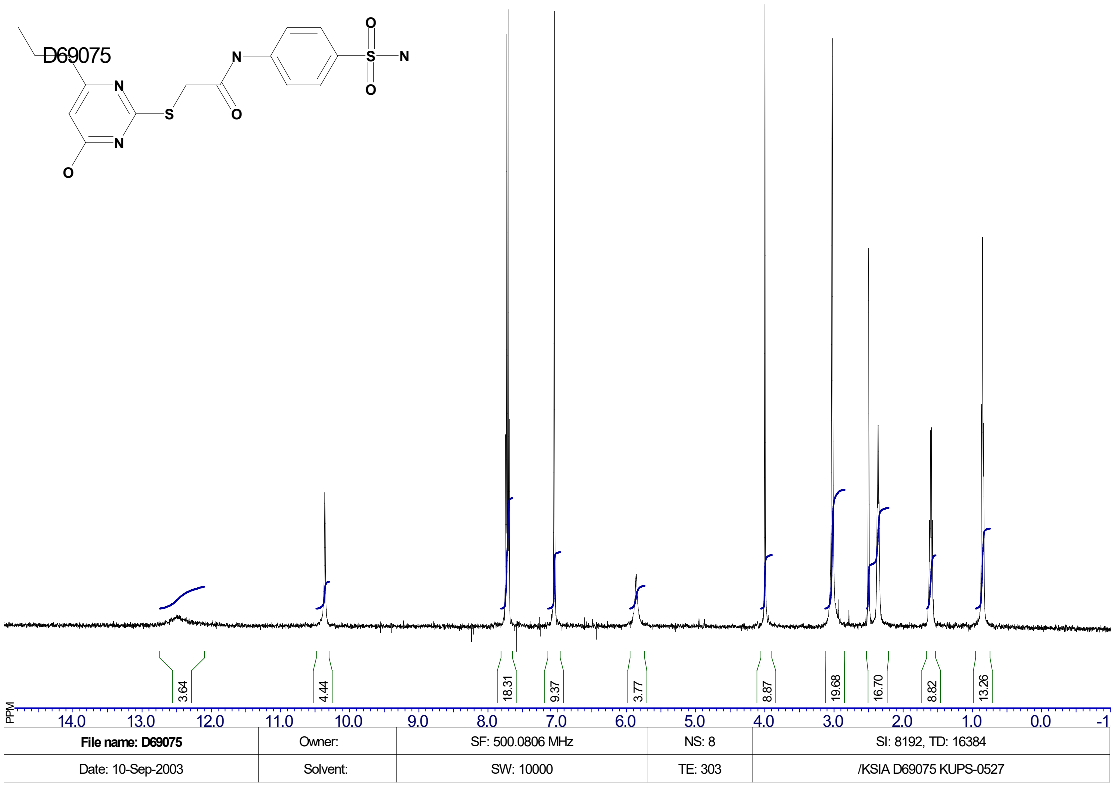
D69075
3.64
4.44
18.31
9.37
3.77
8.87
19.68
16.70
8.82
13.26
PPM
14.0 13.0 12.0 11.0 10.0 9.0 8.0 7.0 6.0 5.0 4.0 3.0 2.0 1.0 0.0 -1
File name: D69075
Owner:
SF: 500.0806 MHz
NS: 8
SI: 8192, TD: 16384
Date: 10-Sep-2003
Solvent:
SW: 10000
TE: 303
/KSIA D69075 KUPS-0527

Compound **18**

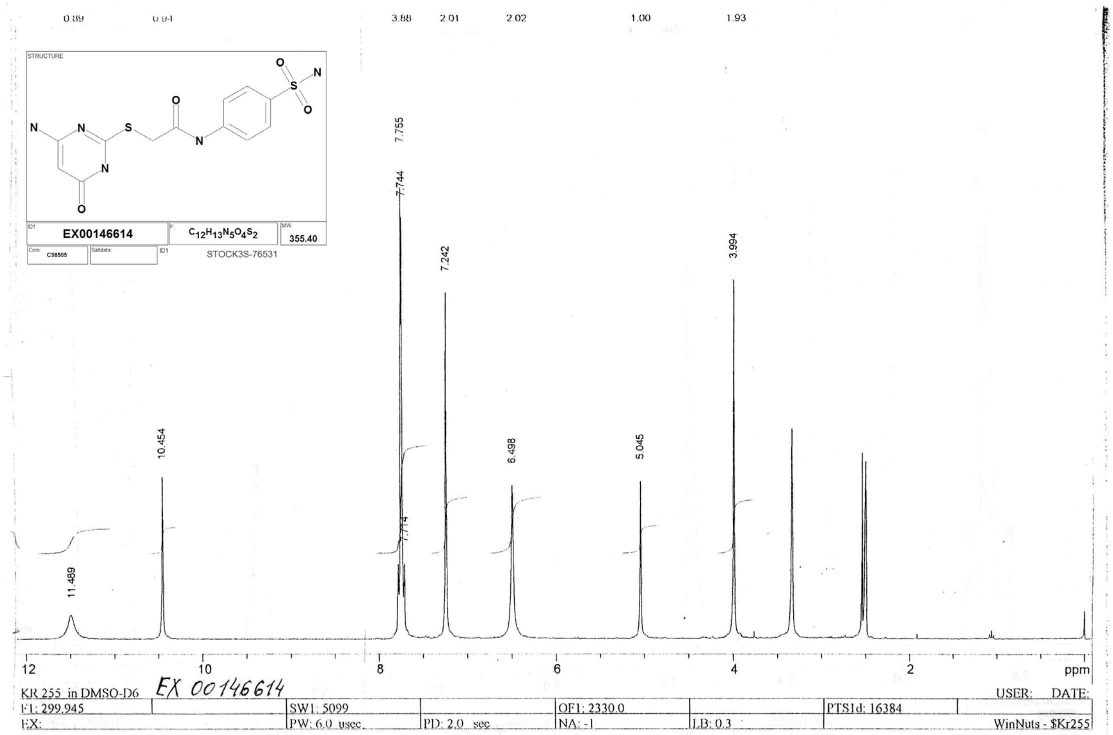

Compound **19**

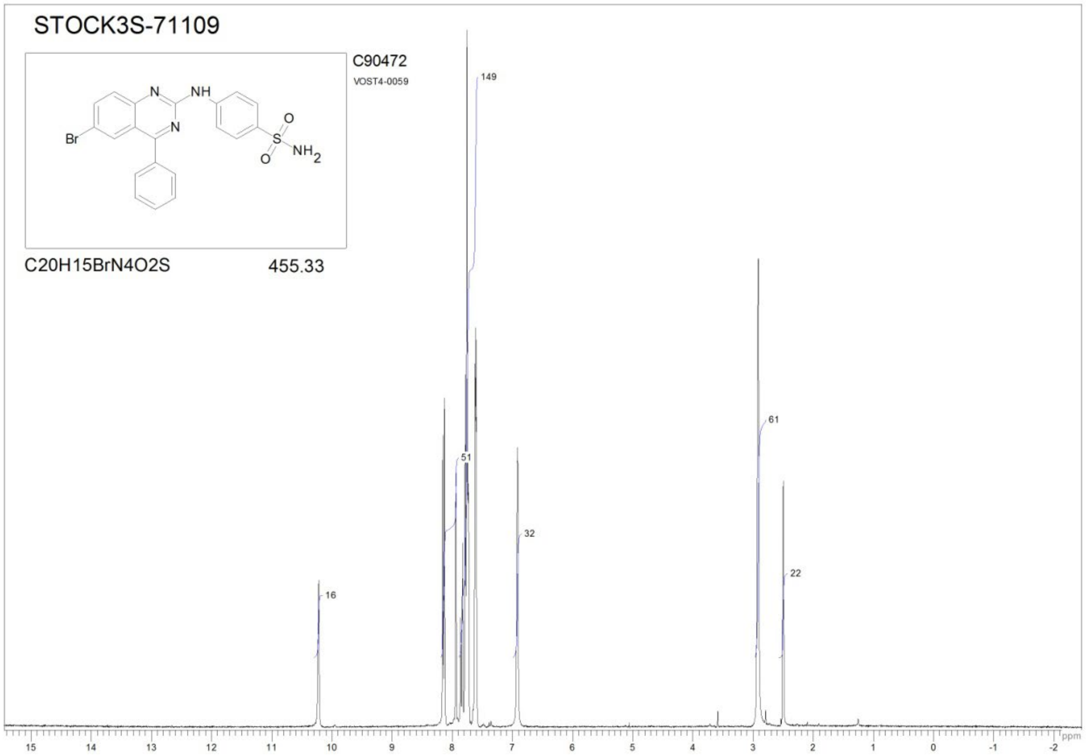

Compound **20**

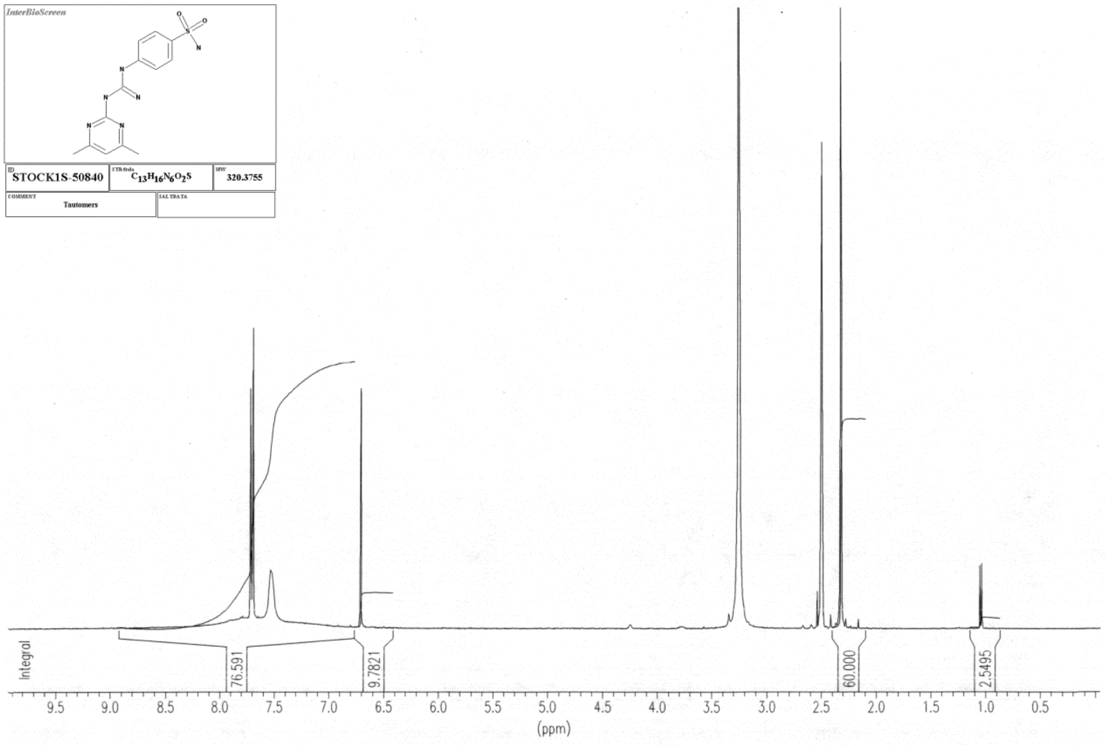

^{13}C-NMR spectra of compounds 2,3

^{13}C-NMR spectrum of ***4-[[(tetrahydro-4,6-dioxo-2-thioxo-5(2H)-pyrimidinylidene)methyl]amino]benzenesulfonamide*** **(2)**

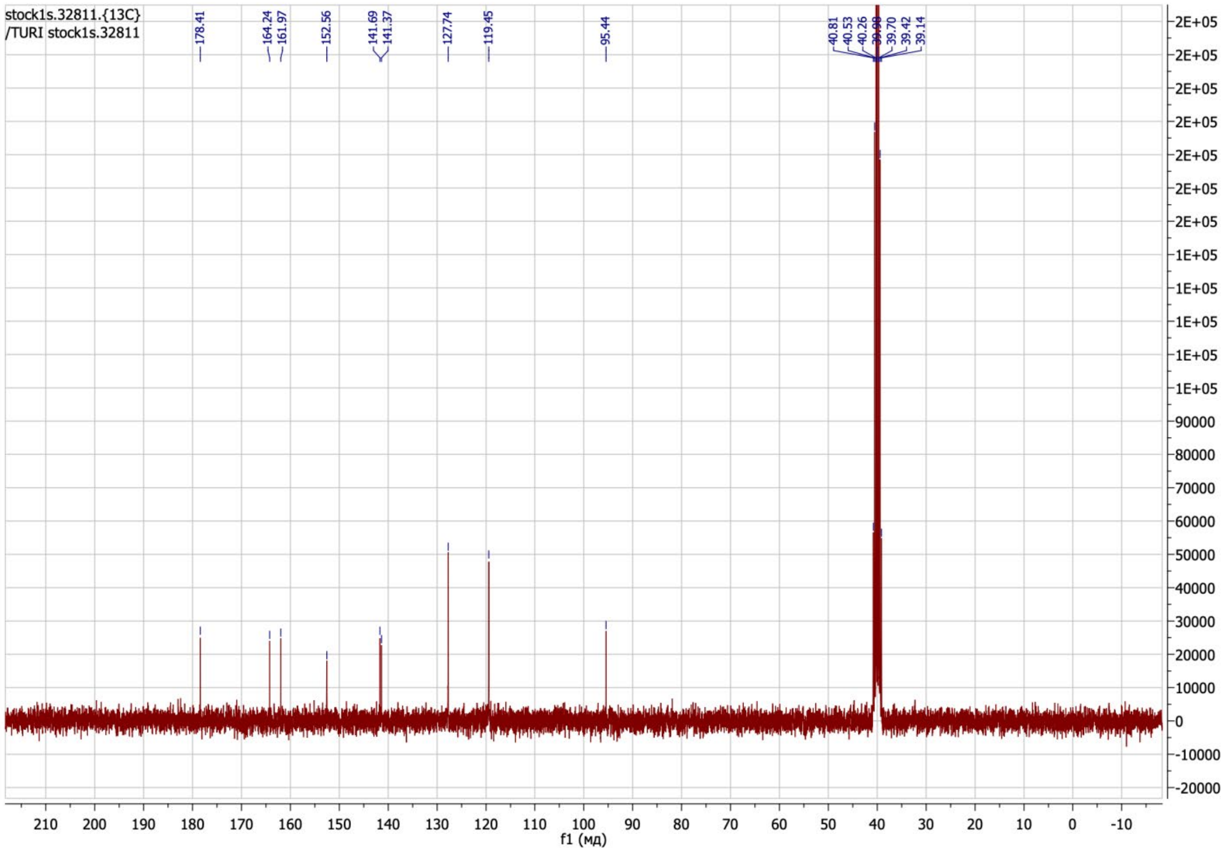

^{13}C-NMR spectrum of ***4-{[(1,3-Dicyclohexyl-2,4,6-trioxotetrahydro-5(2H)-pyrimidinylidene)methyl]amino}benzenesulfonamide (3)***

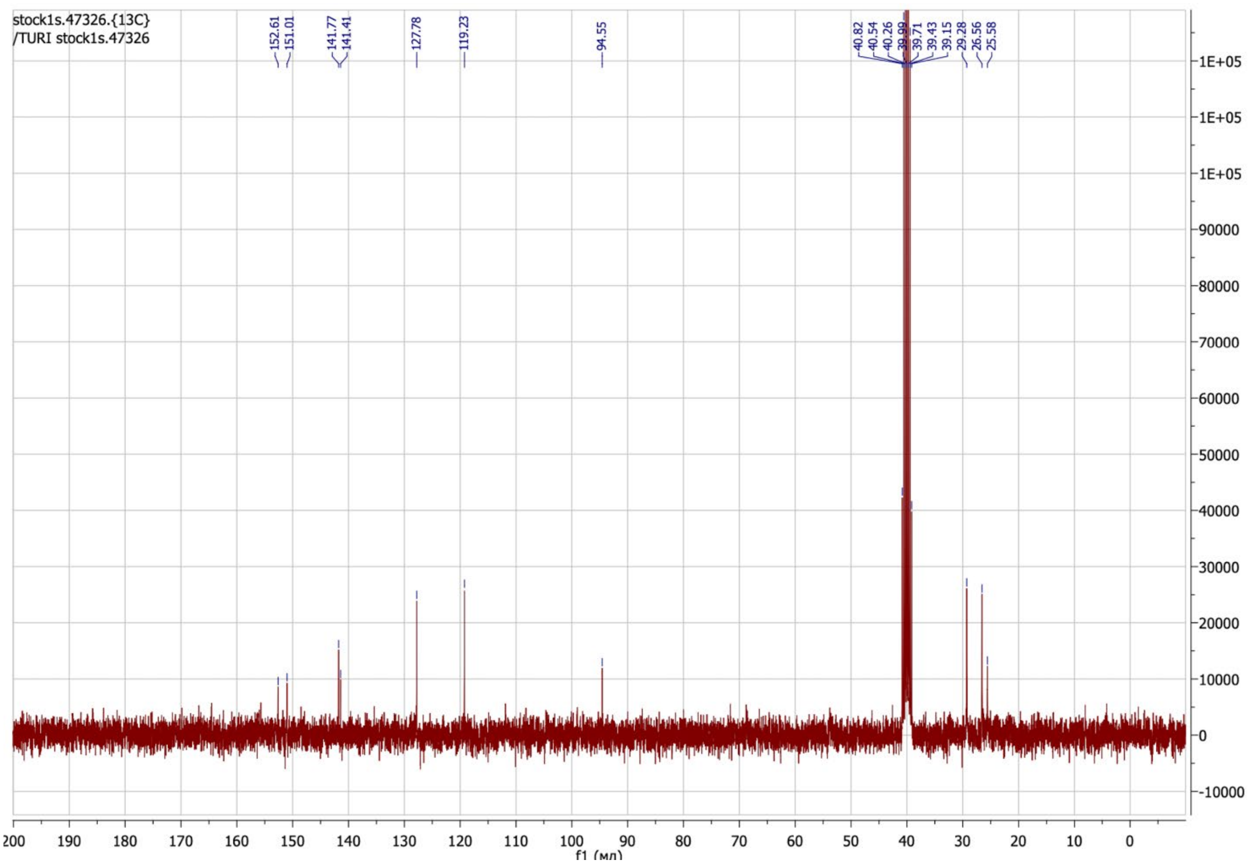

stock1s.47326.{13C}
/TURI stock1s.47326
152.61
151.01
141.77
141.41
127.78
119.23
94.55
40.82
40.54
40.26
39.99
39.71
39.43
39.15
29.28
26.56
25.58
f1 (мд)
